# Methods for conducting systematic reviews of risk factors in low- and middle-income countries

**DOI:** 10.1186/s12874-016-0134-2

**Published:** 2016-03-15

**Authors:** Yulia Shenderovich, Manuel Eisner, Christopher Mikton, Frances Gardner, Jianghong Liu, Joseph Murray

**Affiliations:** Institute of Criminology, University of Cambridge, Sidgwick Ave, Cambridge, CB3 9DA, UK; Department of Violence and Injury Prevention and Disability, World Health Organization, 20 Avenue Appia, 1211 Geneva 27, Switzerland; Department of Social Policy and Intervention, University of Oxford, Barnett House, 32 Wellington Square, Oxford, OX1 2ER UK; School of Nursing, University of Pennsylvania, 418 Curie Blvd, Philadelphia, PA 19104 USA; Postgraduate Programme in Epidemiology, Federal University of Pelotas, Rua Marechal Deodoro, 1160, 3º Piso, CEP: 96020-220 Pelotas, RS Brazil; Department of Psychiatry, University of Cambridge, Douglas House, 18b Trumpington Road, Cambridge, CB2 8AH UK

**Keywords:** Databases, Meta-analysis, Systematic review, Low- and middle-income countries, Conduct problems, Violence

## Abstract

**Background:**

Rates of youth violence are disproportionately high in many low- and middle-income countries [LMICs] but existing reviews of risk factors focus almost exclusively on high-income countries. Different search strategies, including non-English language searches, might be required to identify relevant evidence in LMICs. This paper discusses methodological issues in systematic reviews aiming to include evidence from LMICs, using the example of a recent review of risk factors for child conduct problems and youth violence in LMICs.

**Methods:**

We searched the main international databases, such as PsycINFO, Medline and EMBASE in English, as well as 12 regional databases in Arabic, Chinese, English, French, Spanish, Portuguese and Russian. In addition, we used internet search engines and Google Scholar, and contacted over 200 researchers and organizations to identify potentially eligible studies in LMICs.

**Results:**

The majority of relevant studies were identified in the mainstream databases, but additional studies were also found through regional databases, such as CNKI, Wangfang, LILACS and SciELO. Overall, 85 % of eligible studies were in English, and 15 % were reported in Chinese, Spanish, Portuguese, Russian or French. Among eligible studies in languages other than English, two-thirds were identified only by regional databases and one-third was also indexed in the main international databases.

**Conclusions:**

There are many studies on child conduct problems and youth violence in LMICs which have not been included in prior reviews. Most research on these subjects in LMICs has been produced in the last two-three decades and mostly in middle-income countries, such as China, Brazil, Turkey, South Africa and Russia. Based on our findings, it appears that many studies of child conduct problems and youth violence in LMICs are reported in English, Chinese, Spanish and Portuguese, but few such studies are published in French, Arabic or Russian. If non-English language searches and screening had not been conducted in the current review, 15 % of eligible studies would have been missed. Although there are benefits to non-English language searches and the inclusion of non-English studies in meta-analyses, systematic reviewers also need to consider the resources required to incorporate multi-lingual research.

**Electronic supplementary material:**

The online version of this article (doi:10.1186/s12874-016-0134-2) contains supplementary material, which is available to authorized users.

## Background

Eighty-two percent of the world’s population, including 90 % of 2.2 billion children, live in low- and middle-income countries (LMICs) [1]. Key requirements for responding to the health needs of populations in LMICs is to determine the extent of the problems and identify relevant risk factors [2]. Risk factors are defined as characteristics of people or their environment associated with an increased probability of an adverse outcome [3]. Preventive interventions aim to reduce the occurrence of risk factors and mitigate their impact, as well as to promote protective factors that reduce the probability of adverse outcomes in high risk contexts [4]. In the first survey on mental health research priorities in LMICs, stakeholders and researchers identified prevalence studies and studies of risk factors as the most needed type of research [5]. This paper discusses methods for conducting systematic reviews of risk factor studies in LMICs.

Violence is a major global health problem, and its levels are much higher in many LMICs than in high-income countries (HICs) [6–8]. As adolescence and early adulthood is a peak phase for many problem behaviours [9], the World Health Organization has characterized youth violence as a priority focus area due to its large contribution to the global burden of injury and premature death [10].

Given the high economic and health costs of youth crime and violence, and lack of existing reviews of evidence in LMICs, we conducted a systematic review of correlates and risk factors for perpetrating interpersonal violence and crime among young people 10 to 29 years old. The outcomes of interest included violent and non-violent crime, gang membership, carrying weapons, fighting, and other types of physical violence, such as assault and intimate partner violence. This review defined youth as 10–29 years old, following the World Health Organization definition of youth violence [11]. We also searched for studies examining childhood conduct problems, which often precede youth crime and violence. Severe conduct problems are related to lifelong adverse outcomes, including unemployment, mental health problems, violent and non-violent crime and substance abuse [12–14]. The conduct problems considered in the review were general conduct problems, aggression, bullying, Oppositional Defiant Disorder (ODD) and Conduct Disorder (CD) among children 0 to 18 years old. We excluded studies that examined measures that combined conduct problems with symptoms of attention deficit hyperactivity disorder.

Several extensive reviews have been carried out to examine risk factors for conduct problems, crime and violence in high-income countries [15–18]. While some of the previous reviews did not explicitly exclude research from LMICs, no review we are aware of in this field has actively focused on LMICs. Some reviews even made an explicit decision to focus on only HIC (or “Western”) countries [15]. Most previous reviews have also had relatively exclusive methodological eligibility criteria, which may have resulted in a predominance of studies from HICs. Furthermore, they might not have captured some of the more recent research in LMICs. Therefore, there is a critical need to conduct systematic reviews to synthesise and evaluate evidence on these topics from LMICs [4, 19].

There are particular challenges for conducting systematic reviews aiming to locate and synthesize evidence from LMICs, including the range of languages in which studies may be reported and potential lack of inclusion of study reports in the large international databases usually searched in systematic reviews. An examination of the indexing of publications in psychiatry suggested that the international medical bibliographic databases tend to over-represent US journals compared to European ones [20]. Another examination, focusing on five large Chinese biomedical databases, suggested that less than 6 % of the 2500 journals in those five databases were indexed by MEDLINE [21].

Empirical results from the current review, synthesizing findings on correlates, risk and protective factors will be reported separately in future publications. The current article describes key methodological issues involved in searching for evidence available from LMICs to contribute to “the science of research synthesis” [22]. A specific empirical question we address in this paper is what proportion of the studies found by conducting searches and screening in seven different languages would have been retrieved if we had only searched the main medical and social science databases in English, as is often standard practice in systematic reviews. Are standard practices likely to yield a large proportion of the globally available evidence?

## Methods

### Search strategy

In this systematic review, we aimed to search for all evidence on risk factors for childhood externalising behaviours and youth crime and violence in LMICs. Drawing on a combination of free-text search terms, Medical Subject Headings and database-specific subject headings, we developed a sensitive search strategy for multiple electronic databases (see Additional file 1). The key concepts that orientated the search procedures were “low- and middle-income countries”, “children and youth”, and “conduct problems, crime and violence”. It is considered essential to search multiple international databases for a systematic review [23], particularly in a cross-disciplinary area of research [24]. In August-September 2013 we searched PsycINFO, MEDLINE, EMBASE, CINAHL, EconLit, Criminal Justice Abstracts, Russian Academy of Sciences Bibliographies, Sociological Abstracts & Social Services Abstracts, Applied Social Sciences Index and Abstracts, International Bibliography of the Social Sciences, ERIC, Web of Science, Global Health Library, National Criminal Justice Reference Service Abstracts Database, CENTRAL, JOLIS, World Bank, Open Grey, and Google Scholar, without restrictions on study years or languages. The searches of these databases were conducted in English.

To complement the searches of key international databases, we also used non-English-language search terms to examine 12 regional databases (see Table 1). The regional databases were identified by foreign language team members, and from the special issue of Emerging Themes in Epidemiology: “Beyond English: Accessing the global epidemiological literature”. As suggested by Fung [25] in that journal issue, we focused our searches on the official languages of the United Nations: Arabic, Chinese, English, French, Spanish and Russian, and also added Portuguese. Some of the non-English databases we worked with do not allow complex search strategies, so due to the nature of database interfaces the searches in these databases were less systematic. We used search terms for the behavioural outcomes of interest and, where possible, terms for children and youth. We screened all individual search results wherever possible. In several cases, where the search yielded an unmanageable number of hits, we screened pages with results until the titles appeared irrelevant, based on the searcher’s subjective judgement. Hence the figure of relevant publications in those databases may be somewhat underestimated. Furthermore, the foreign language team members searched Google Scholar and used internet search engines to look for relevant studies and reports. We have also identified studies through reference lists of included publications (reference harvesting). In addition, about 200 researchers and organizations were contacted by email to identify unpublished studies.

**Table 1:** Databases searched with non-English terms

Language	Databases
Arabic	Index Medicus for the Eastern Mediterranean Region
	King Saud University Repository
	YU-DSpace Repository
Chinese	Cnki
	Wanfang Data
	Cqvip
French	Index Medicus Afro
	Revue de Médicine tropicale
	Agence Universitaire de la Francophonie
	Refdoc
Russian	Elibrary.ru
	Panteleimon
Spanish and Portuguese	LILACS
	SciELO

Note that LILACS and SciELO were searched using English as well as Spanish and Portuguese search terms to fully utilize the databases

## Results

### Inclusion criteria

The review protocol was made public online in advance of the review. A full description of study selection criteria can be found in a supplementary online file (see Additional file 2). In brief, to be eligible, studies has to be conducted in a LMIC, and report an estimate of the strength of association between an eligible outcome and a potential correlate, or enough numerical information to calculate an association. The correlate and behavioural outcome had to be measured at the level of the individual. For example, studies at the neighborhood level comparing rates of violence across neighbourhoods with different levels of poverty were excluded. To restrict the review to more population-based generalizable studies, we only included studies with at least 100 participants recruited through random, stratified probability, or total population sampling within households, or two or more institutions in the community, such as schools, or maternity hospitals in birth cohort studies. Although we were most interested to review evidence on risk factors, which by definition precede the outcome and should be evaluated in longitudinal studies, results on correlates measured in cross-sectional studies were also eligible for the review because we did not expect to find many eligible longitudinal studies in LMICs. We also included case–control studies, for instance comparing a group of people in a young offenders’ institution and a matched group of youth in the general population.

### Searching and screening

**Fig. 1:**
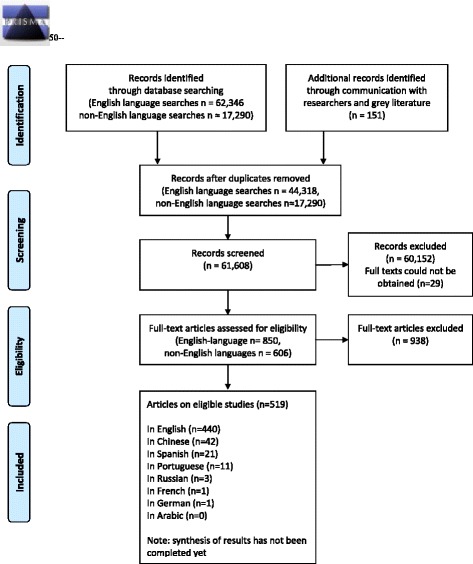
PRISMA Flow Diagram

Our searches produced a total of 79,786 titles (see Fig. 1), including 62,346 records identified from the English language searches and reference harvesting, 17,290 records identified from non-English language searches and about 150 records identified from communication with researchers and grey literature. In addition to the results from regional databases, the international databases also produced a number of titles in languages other than English. Initially, all titles and abstracts were examined by the first author, and the texts of 1437 English-language studies that seemed possibly relevant to the review or did not have enough information to determine relevance from the abstract were retrieved for a full-text screening. We note that, given the very large number of studies screened for this review, due to limited resources the screening was performed by one researcher (first author), in consultation with the corresponding author in cases of uncertainty. Searches in non-English languages were conducted by graduate students (either native speakers or speaking the search language fluently). Working with the first author, the people working in non-English languages screened the titles, abstracts and 605 full-texts to identify eligible studies. For eligible studies, the key parts of the non-English articles (setting, methods, and results) were translated into English for inclusion in the review.

After screening with a specially designed form (see Additional file 3), 519 articles were identified as eligible for the review. Eligible studies may have included more than one eligible outcome for the review. Most of the eligible studies included in the review were English-language studies identified in the mainstream databases. Most of these were peer-reviewed academic journal publications, and 11 dissertations. Grey literature searches beyond indexed databases made a small contribution to this review, including ten additional reports (five from South Africa and one each from Brazil, Chile, China, Mexico, and Jamaica) not published in academic journals at the time but identified by email communication or internet searches. Ten additional publications were identified in reference harvesting (references from other included studies). Finally, 11 publications were chapters of the book describing the Second International Self-Report Delinquency Study, including the only studies identified from Armenia, Slovenia and Surinam.

Overall, 85 % of eligible studies were in English, and 15 % were reported in Chinese, Spanish, Portuguese, Russian, French, or German and translated to English for use in the review. Of the 77 studies reported in Chinese, Spanish, Portuguese, Russian and French, 25 eligible texts were identified both by well-known subscription databases, such as PsycINFO, Medline or EMBASE, as well as regional databases. Therefore, the regional database searches in foreign languages identified an additional 52 publications. Of these, the Chinese databases CNKI and Wangfang located 37 additional unique eligible studies, SciELO search identified seven, LILACS a further five, and elibrary.ru another three texts. The studies included from the regional databases report on research from China (37), Brazil (4), Russia (3), Colombia (3), Chile (2), Peru (2), and Mexico (1). Thus, most of the studies identified in the regional databases described research from countries also represented in the international databases – with the exception of Peru, as the only two publications available from there came from Lilacs. Finally, we also identified 39 potentially eligible articles in languages that we did not have the resources to screen in full-text (13 in Polish, 9 in Croatian, Serbian and Bosnian, 8 in Turkish, 4 in Hungarian, 4 in Lithuanian, and 2 in Persian). We were able to include one longitudinal study in German, identified through mainstream databases, but did not carry out any German-language searches.

### Included study characteristics

Most of the studies identified by the review were based on cross-sectional studies (442). The review also included 76 reports on longitudinal studies, in which the risk factor preceded the outcome. Correlates and risk factors reported included, among others, the gender and age of the child/young person, psychological characteristics, substance use, health indicators, school performance, parenting factors such as parenting styles and attitudes, family socioeconomic status, and urban versus rural residence. An individual eligible study could include results on several relevant outcomes. Of the 351 studies of 0–18 year olds, 179 included results for general conduct problems, 146 for aggression, 35 for bullying perpetration and 16 for CD/ODD. Among 174 studies focusing on 10–29 year olds, 72 studies examined fighting, 45 carrying weapons, 48 other violence, 61 combined measures of non-violent and violent crime, 9 sexual or intimate partner violence and 8 gang membership.

**Fig. 2:**
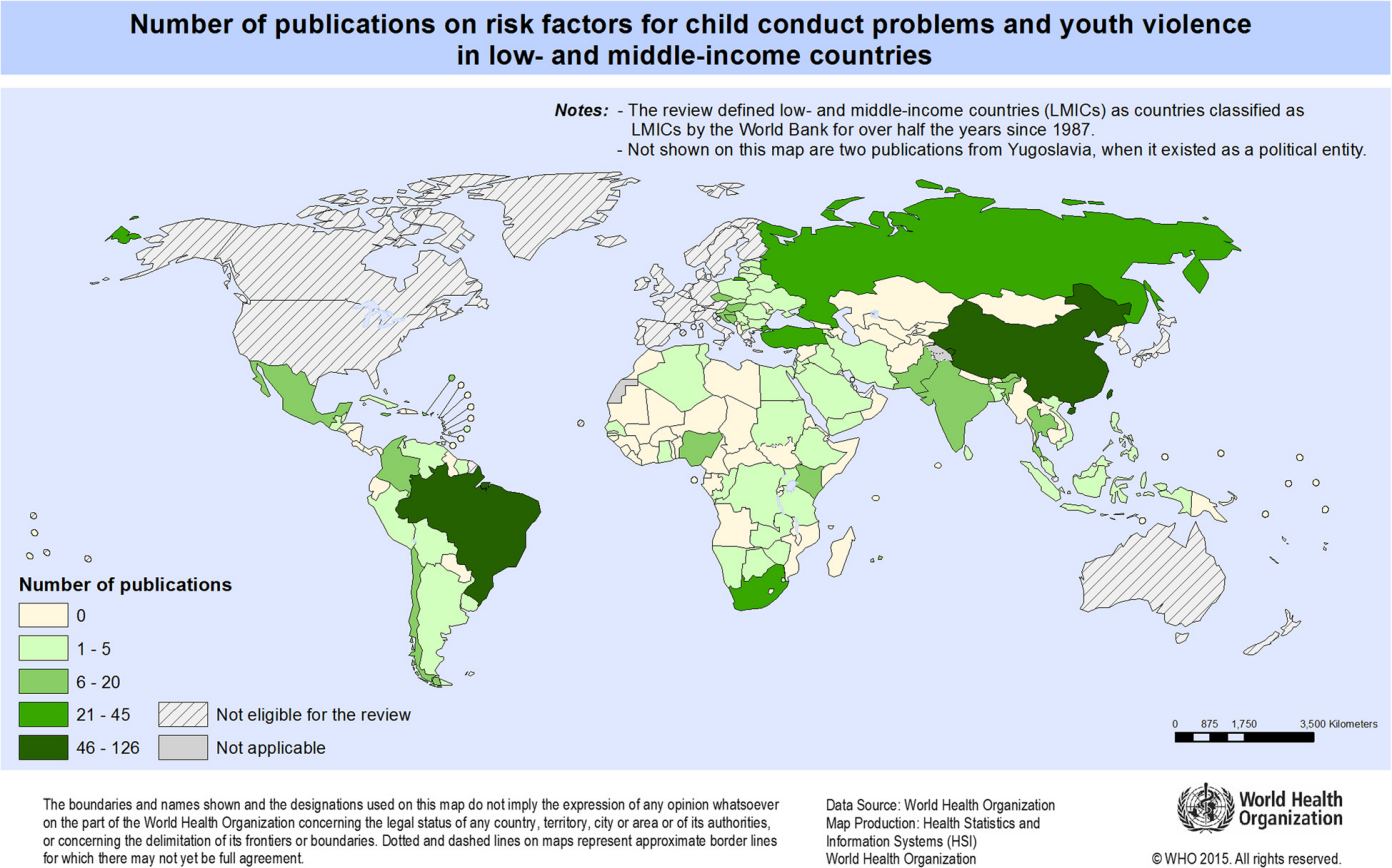
Number of included publications, by country of the research

China contributed the largest number of studies to this review (124), followed by research based in Brazil (46), Turkey (33), South Africa (29) and Russia (23) following. Several other LMICs with large populations, such as India (12), Nigeria (8), Pakistan (6), Bangladesh (3) and Indonesia (1), only contributed a few studies to the review. Overall, 81 countries have contributed to our database (see the map in Fig. 2 for a full overview).

**Fig 3:**
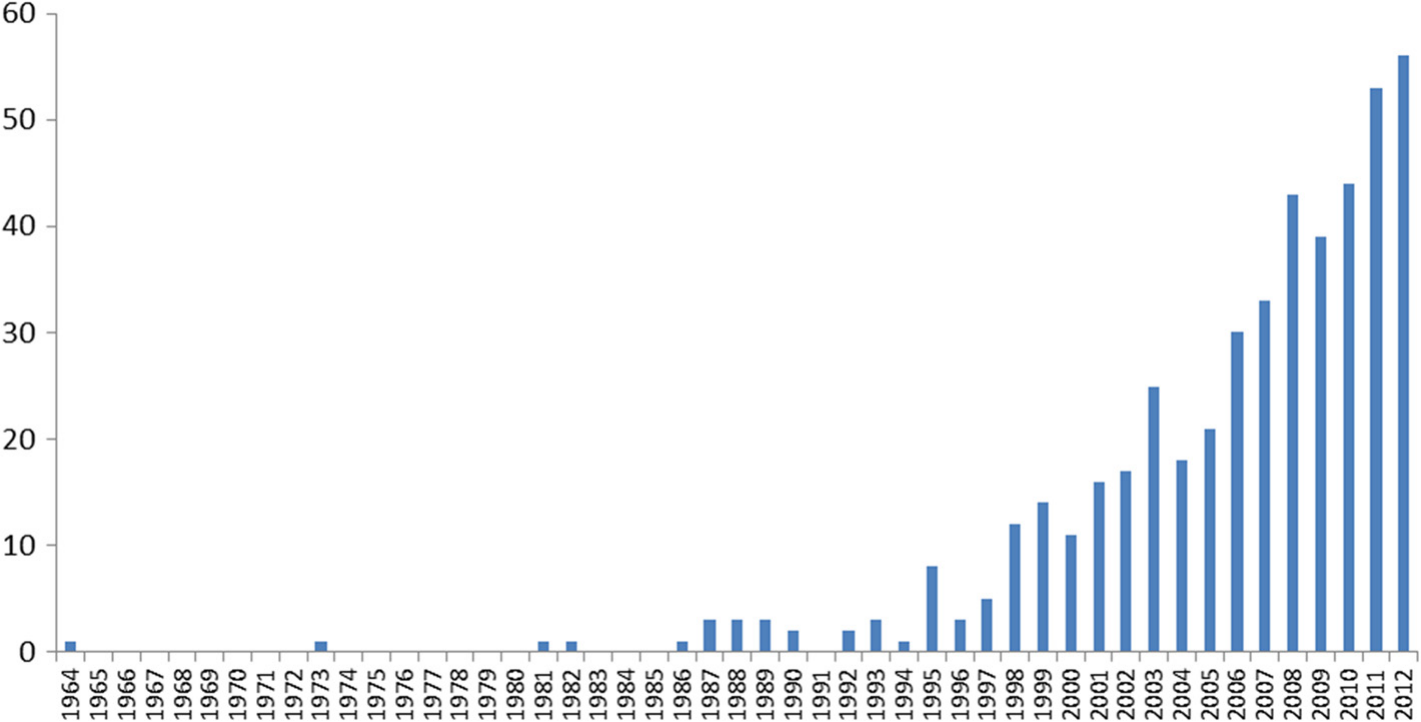
Number of included publications, by publication year

Among the included studies, 86 % were published since 2000, and only 14 papers (3 %) were published pre-1990 (see Fig. 3), suggesting that most research on these issues in LMICs has been carried out in the last two-three decades. It is also possible that earlier research is not as accessible online.

As the included studies have not been fully coded yet, we cannot assess their quality. From the full-text screening, we know that a number of studies did not clearly specify the ages of participants, only providing the mean age of the sample or their stage of education (e.g., “secondary school students”). Clear information on sampling strategy was missing in 30 % of included studies. We chose to include such studies in our review but coded that sampling was “not clear”. In particular, we note that most of the reports in Chinese tended to be brief (3–4 pages), and many did not contain some important information, such as the sampling strategy.

## Discussion

Even with several restrictions on study design, such as a minimum sample size of 100 participants in community samples, we found a large number of studies providing evidence on correlates of childhood conduct problems, and, to a smaller extent, youth crime and violence in LMICs.

The publication dates of eligible studies retrieved for our review show that an increasing number of studies are being carried out in LMICs. In our review, 86 % of eligible studies were published since 2000. Our findings also suggest that only a few relevant studies have been carried out in some LMIC regions, such as South Asia. The locations of included studies suggest that the bulk of LMIC evidence is produced in several middle-income countries, such as China and Brazil, and very little in low-income countries. Although it is possible that different search methods might have located more studies in other parts of the world, this finding is similar to that of other reviews in international mental health literature [19, 26, 27].

Studies of language bias suggest that the majority of systematic reviews tend to only include studies in English [28]. We explored a number of regional databases in Spanish, Portuguese, Chinese, French, Arabic and Russian that uniquely hosted 10 % of the eligible studies. In addition, we screened and included studies in languages other than English, which were identified in the mainstream databases (5 % of the eligible studies). Combined, foreign language studies accounted for 15 % of the total. Hence, if the non-English language searches and screening had not been conducted in the current review, 15 % of eligible studies would have been missed. Based on our findings, it appears that many studies examining child conduct problems and youth violence in the areas of child psychology, psychiatry and criminology in LMICs are reported in Chinese, Spanish and Portuguese, but few studies are likely to be reported in French, Arabic or Russian (although there is a number of studies from Russia in English). The project is currently at the stage of coding and analyses of the eligible studies, so it is not yet possible to determine whether the inclusion of non-English studies will impact the findings.

The influence of only searching in English has been examined in relation to systematic reviews of intervention evaluations. Two examinations of pairs of RCT reports in German and English suggested that programme evaluations with significant results were more likely to be published in English language journals than in non-English language journals [29, 30], suggesting that multi-lingual searches are needed to prevent bias. However, several other retrospective analyses of systematic reviews suggested that excluding non-English trials would not have substantially altered review conclusions [31, 32]. Including studies in languages other than English is clearly advantageous to increase the number of studies in a review, and thereby increase statistical power in meta-analyses, and external validity of the evidence. Further research is required concerning the potential impact of including articles from non-English journals on the power of the meta-analyses as well as the potential systematic bias introduced by only searching and screening in English.

Researchers also need to consider the resources required to incorporate multi-lingual research. In particular, to carry out screening and selection of studies that are not in English, team members are required who are fluent in those languages, and they need to be either fully familiar with the selection criteria, or translate study methods for others to evaluate, which can require considerable resources or decrease consistency in study screening. Hence, reviewers need to balance the use of resources with the gains made in terms of the number of additional eligible studies found by searching in languages other than English. It has been proposed that, for randomized trials, initial eligibility screening might be carried out even without knowledge of the relevant language, using English-language abstracts, and indicators such as the number of authors listed and presence of a CONSORT participant flowchart [33].

Most of our efforts in this review focussed on searching for published studies, but we also contacted over 200 researchers and practitioners to try to locate grey literature, and used Google Scholar to search for unpublished reports. Grey literature is considered important to minimize publication bias [34], although several researchers suggest that non-peer reviewed studies tend to be lower quality than studies published in peer reviewed journals [35]. It is possible that other ways of searching for grey literature are necessary, to find high quality studies of correlates and risk factors in non-peer-reviewed reports. For example, researchers may consult studies carried out by international organizations, such as Global school-based student health survey coordinated by the World Health Organization. Although such reports may not directly examine risk factors, correlates can be calculated from the available data.

Future reviews of studies in LMICs could make valuable contributions by further investigating the proportion of studies identified in different databases. An extensive list of regional LMIC databases has been created by the Norwegian Satellite of the Cochrane Collaboration Effective Practice and Organisation of Care Group.^1^ Furthermore, there are a growing number of databases collecting impact evaluations of programmes and policies in LMICs. Extensive lists of relevant databases have been collected by the Campbell Collaboration International Development Coordinating group^2^ and the Center for Global Development.^3^

Guidelines for search strategies for observational studies are much less developed than for identifying evaluation studies such as randomized trials [36]. Campbell Collaboration guidelines recommend that “High sensitivity should be sought, which may result in relatively low precision” [37]. However, in a large project this may lead to an overwhelming number of citations. We believe our searches were very sensitive, but certainly not precise: less than 1 % of the unique database citations screened was eventually included in the review. For comparison, Sampson et al. [38] calculated that a sample of 94 MEDLINE-indexed systematic reviews had 3 % precision on average, although there was a wide range of precision in the reviews. It may be difficult to maintain sensitivity and identify all relevant studies that include correlates or risk factors with a more specific search strategy. We had considered using additional search terms for study design for example, but decided not to do so in order to maximise the chances of locating relevant studies. As part of developing guidance for future reviews of risk and protective factors, it would be useful to propose peer-reviewed search filters for studies of correlates.

## Conclusions

There are many studies on conduct problems and youth crime and violence in LMICs which have not been included in prior reviews, which, for various reasons, have focused on HICs. However, most research on these subjects in LMICs has been produced in a handful of countries. Searches in languages other than English, especially in Chinese, Spanish and Portuguese, as well as the use of less well-known regional databases, such as CNKI, Wangfang, LILACS and SciELO, can contribute to systematic reviews of risk factor studies in LMICs. Future systematic review guidance should include advice on identifying and including studies reported in languages other than English.

## Additional files

Additional file 1: Detailed review search strategy. The file includes the names of databases used for this systematic review and the search strategies used for each database. (DOCX 105 kb) Additional file 2: Review protocol. The file includes the protocol for this systematic review, also published at http://www.vrc.crim.cam.ac.uk/vrcresearch/meLMIC/protcolriskfactors. (PDF 748 kb) Additional file 3: Study screening tool. The file includes a detailed screening tool used for selecting studies into the systematic review. (DOCX 31 kb)

## Abbreviations

ODD: Oppositional Defiant Disorder
CD: Conduct Disorder
LMICs: low- and middle-income countries
HICs: high-income countries

## Footnotes

1 http://epoc.cochrane.org/sites/epoc.cochrane.org/files/uploads/LMIC%20Databases%20August%202013.pdf

2 http://www.campbellcollaboration.org/artman2/uploads/1/Database_Guide_for_SRs_in_International_Development_.pdf

3 www.cgdev.org/sites/default/files/IE%20database_final.pdf
