# Supplementary material for: Methods for conducting systematic reviews of risk factors in low- and middle-income countries
Source: BMC Med Res Methodol. 2016 Mar 15;16:32. doi: 10.1186/s12874-016-0134-2 (PMC4791911; doi:10.1186/s12874-016-0134-2)
Supplement: Additional file 1: — Detailed review search strategy. The file includes the names of databases used for this systematic review and the search strategies used for each database. (DOCX 105 kb) [file 12874_2016_134_MOESM1_ESM.docx]

Databases searched using English-language terms (hosting platforms in brackets):

| Database | Titles |
| --- | --- |
| EMBASE (Ovid) 1974 to 2013 Week 35 | 14260 |
| Ovid MEDLINE(R) In-Process & Other Non-Indexed Citations and Ovid MEDLINE(R) 1946 to Present | 11842 |
| Web of Science | 6248 |
| PsycINFO (Ovid) 1967 to 2013 | 4480 |
| Criminal Justice Abstracts (EBSCOHost) | 4168 |
| Sociological Abstracts + Social Services Abstracts (ProQuest) | 3404 |
| CINAHL (EBSCOhost) | 3052 |
| International Bibliography of the Social Sciences (IBSS) (ProQuest) | 1687 |
| ERIC (ProQuest) | 1325 |
| World Bank | 1236 |
| Applied Social Sciences Index and Abstracts (ProQuest) | 801 |
| EconLit (EBSCOhost) | 124 |
| National Criminal Justice Reference Service Abstracts Database | 91 |
| JOLIS (IMF, World Bank and International Finance Corporation) | 80 |
| Russian Academy of Sciences Bibliographies (EBSCOHost) | 68 |
|  |  |
|  |  |

Non-English databases

| Language | Databases | Titles reviewed |
| --- | --- | --- |
| Arabic | Index Medicus for the Eastern Mediterranean Region | 0 |
|  | King Saud University Repository | 275 |
|  | YU-DSpace Repository | 185 |
| Chinese | Cnki | 1360 |
|  | Wanfang Data | 2120 |
|  | Cqvip | 580 |
| French | Index Medicus Afro | 29 |
|  | Revue de Médicine tropicale | 558 |
|  | Agence Universitaire de la Francophonie | 0 |
|  | Refdoc | 338 |
| Russian | Elibrary.ru | 2366 |
| Spanish and Portuguese | Lilacs *including in English* | 8128 |
|  | Scielo *including in English* | 1352 |
| Total |  | 17291 |

**Search strategy structure**

A AND B AND D

or

C AND D

**CONCEPT A**

- child
- youth
- infant
- baby
- toddler
- adolescent
- teenager

**CONCEPT B**

aggression

antisocial behaviour

behavior disorder

behavior problem

bullying

conduct disorder

conduct problem

crime

criminal behavior

disruptive behaviour disorder

externalising

externalizing

gang

homicide

oppositional defiant disorder

school violence

# social behavior disorders

violence

violent crime

workplace violence

**CONCEPT C**

- juvenile delinquency
- child behavior disorders
- school violence

**CONCEPT D**

- Africa or Central Africa or Latin America or Caribbean or West Indies or Eastern Europe or Soviet or South America or Arab or Middle East or Latin America or Central America
- Afghanistan or Albania or Algeria or Angola or Antigua or Barbuda or Argentina or Armenia or Armenian or Aruba or Azerbaijan or Bahrain or Bangladesh or Barbados or Benin or Byelarus or Byelorussian or Belarus or Belorussian or Belorussia or Belize or Bhutan or Bolivia or Bosnia or Herzegovina or Hercegovina or Botswana or Brasil or Brazil or Bulgaria or Burkina Faso or Burkina Fasso or Upper Volta or Burundi or Urundi or Cambodia or Khmer Republic or Kampuchea or Cameroon or Cameroons or Cameron or Camerons or Cape Verde or Central African Republic or Chad or Chile or China or Colombia or Comoros or Comoro Islands or Comores or Mayotte or Congo or Zaire or Costa Rica or Cote d'Ivoire or Ivory Coast or Croatia or Cuba or Cyprus or Czechoslovakia or Czech Republic or Slovakia or Slovak Republic or Djibouti or French Somaliland or Dominica or Dominican Republic or East Timor or East Timur or Timor Leste or Ecuador or Egypt or United Arab Republic or El Salvador or Eritrea or Estonia or Ethiopia or Fiji or Gabon or Gabonese Republic or Gambia or Gaza or Georgia Republic or Georgian Republic or Ghana or Gold Coast or Greece or Grenada or Guatemala or Guinea or Guam or Guiana or Guyana or Haiti or Honduras or Hungary or India or Maldives or Indonesia or Iran or Iraq or Isle of Man or Jamaica or Jordan or Kazakhstan or Kazakh or Kenya or Kiribati or Korea or Kosovo or Kyrgyzstan or Kirghizia or Kyrgyz Republic or Kirghiz or Kirgizstan or Lao PDR or Laos or Latvia or Lebanon or Lesotho or Basutoland or Liberia or Libya or Lithuania or Macedonia or Madagascar or Malagasy Republic or Malaysia or Malaya or Malay or Sabah or Sarawak or Malawi or Nyasaland or Mali or Malta or Marshall Islands or Mauritania or Mauritius or Agalega Islands or Mexico or Micronesia or Middle East or Moldova or Moldovia or Moldovian or Mongolia or Montenegro or Morocco or Ifni or Mozambique or Myanmar or Myanma or Burma or Namibia or Nepal or Netherlands Antilles or New Caledonia or Nicaragua or Niger or Nigeria or Northern Mariana Islands or Oman or Muscat or Pakistan or Palau or Palestine or Panama or Paraguay or Peru or Philippines or Philipines or Phillipines or Phillippines or Poland or Portugal or Puerto Rico or Romania or Rumania or Roumania or Russia or Russian or Rwanda or Ruanda or Saint Kitts or St Kitts or Nevis or Saint Lucia or St Lucia or Saint Vincent or St Vincent or Grenadines or Samoa or Samoan Islands or Navigator Island or Navigator Islands or Sao Tome or Saudi Arabia or Senegal or Serbia or Montenegro or Seychelles or Sierra Leone or Slovenia or Sri Lanka or Ceylon or Solomon Islands or Somalia or South Africa or Sudan or Suriname or Surinam or Swaziland or Syria or Tajikistan or Tadzhikistan or Tadjikistan or Tadzhik or Tanzania or Thailand or Togo or Togolese Republic or Tonga or Trinidad or Tobago or Tunisia or Turkey or Turkmenistan or Turkmen or Uganda or Ukraine or Uruguay or USSR or Soviet Union or Union of Soviet Socialist Republics or Uzbekistan or Uzbek or Vanuatu or New Hebrides or Venezuela or Vietnam or Viet Nam or West Bank or Yemen or Yugoslavia or Zambia or Zimbabwe or Rhodesia
- LMICs
- developing/less developed/under developed/underserved/deprived/poor countries
- transitional countries

**MeSH terms:**

developing countries

juvenile delinquency

child behavior disorders

social behavior disorders

conduct disorder

aggression

crime

bullying

homicide

child

infant

child health services

child welfare

child behavior

child care

child development

| **Arabic** | Child  طفل  Youth  شباب  Infant  رضيع  Baby  طفل رضيع  Toddler  طفل صغير  Adolescent / Teenager  مراهق  Child development  تنمية الطفل / تطور الطفل  Adolescent development  تنشئة المراهقين  Conduct problems  مشاكل سلوكية  Conduct disorders  اضطرابات سلوكية  Oppositional defiant disorder  خلل التحدي الاعتراضي  Aggression  العدوانية/ الاعتداء  Bullying  البلطجة/ تنمر  Homicide  قتل  Crime  جريمة  Gang  عصابة  Violence  عنف  Social behaviour disorders  اضطرابات السلوك الاجتماعي  Criminal behaviour  السلوك الإجرامي  Violent crime  الجرائم العنيفة  Antisocial behaviour  سلوك غير اجتماعي/ تصرف معادي للمجتمع  Behaviour problem  المشاكل السلوكية  Behaviour disorder  اضطرابات سلوكية  School violence  العنف في المدارس / العنف المدرسي  Workplace violence  العنف في مكان العمل | | | | Disruptive behaviour disorder  اضطراب التصرفات التخريبية  Juvenile delinquency  جنوح الأحداث / انحراف الأحداث  Child behaviour disorders  اضطرابات سلوك الطفل  School violence  العنف في المدارس / العنف المدرسي  Egypt  مصر  Sudan  السودان  Algeria  الجزائر  Morocco  المغرب  Iraq  العراق  Saudi Arabia  المملكة العربية السعودية  Yemen  اليمن  Syria  سوريا  Tunisia  تونس  Chad  تشاد  Somalia  الصومال  Libya  ليبيا  Jordan  الأردن  Eritrea  إريتريا  United Arab Emirates  الإمارات العربية المتحدة  Palestine  فلسطين  Lebanon  لبنان  Kuwait  الكويت  Mauritania  موريتانيا  Oman  عمان  Qatar |
| --- | --- | --- | --- | --- | --- |
|  | قطر  Djibouti  جيبوتي  Bahrain  البحرين  Comoros  جزر القمر | |  | | |
| **Chinese** | Cbullying  欺负问题  bullying / school violence  校园霸凌 / 校园暴力  child behaviour disorder  儿童行为问题  child conduct problems  儿童品行问题 / 品行问题  child conduct problems + risk factors  儿童品行问题 + 危险因素  conduct disorder  品行障碍  juvenile delinquency  青少年违法 / 少年违法  juvenile delinquency + factors  青少年违法 + 危险因素  juvenile delinquency + risk factors  青少年犯罪 + 危险因素  school violence  校园欺凌问题/校园暴力  school violence risk factors  校园暴力危险因素  young people aggression  少年攻击行为 |  | | | |
| **French** | *Adolescent*  adolescent  *école*  school  *enfant*  child  *jeune*  young  *juvenile*  juvenile  *comportement*  behavior  *conduite*  conduct  *crime*  crime  *gang*  gang  *harcèlement/ mobbing*  harassment / bullying  *homicide*  homicide  *trouble oppositionnel avec provocation*  Oppositional Defiant Disorder  *violence*  violence  *risqué*  risky  *délinquance*  delinquency  *délinquance juvenile*  juvenile delinquency  *Afrique*  Africa  *Congo*  Congo  *Côte d'Ivoire*  Côte d'Ivoire  *Cameroun*  Cameroon | | |  | |

| **Portuguese** | *criança*  *lactente*  *adolescente*  *psiquiatria infantil*  *comportamento infantil*  *comportamento do adolescente*  *desenvolvimento do adolescente*  *comportamento do adolescente*  *quadrilha*  *crime*  *comportamento anti-social*  *violência*  *bullying*  *agressão*  *homicídio*  *violência doméstica*  *transtornos do*  *comportamento*  *transtornos do comportamento social*  *transtorno da Conduta*  *transtorno desafiador de oposição*  *transtorno desafiador-opositivo*  *transtornos do comportamento infantil*  *delinquência juvenil* |  | |
| --- | --- | --- | --- |
| **Russian** | *ребенок*  *дети*  *молодежь*  *младенец*  *ребенок*  *подросток*  *буллинг*  *агрессивное поведение*  *расстройство поведения*  *антисоциальное поведение*  *диссоциальное поведение*  *насилие*  *оппозиционно-вызывающее поведение*  *вызывающее оппозиционное расстройство*  *оппозиционно-вызывающее расстройство*  *оппозиционное вызывающее расстройство*  *оппозиционное расстройство неповиновения*  *преступность несовершеннолетних расстройства социального поведения*  *экстернализация*  *банда*  *преступность несовершеннолетних*  *насилие в школе* | |  |

| **Spanish** | *Niño*  *lactante*  *adolescente*  *psiquiatría infantil*  *conducta infantil*  *conducta del adolescente*  *desarrollo del adolescente*  *conducta del adolescente*  *pandilla*  *crimen*  *conducta anti-social*  *violencia*  *acoso escolar*  *agresión*  *homicidio*  *violencia doméstica*  *transtorno da conduta*  *trastorno de la conducta*  *social*  *trastorno del comportamiento*  *trastorno desafiante por oposición*  *trastorno de oposición desafiante*  *transtorno da personalidade anti-social*  *trastornos de la conducta infantil*  *delincuente*  *delincuentes*  *delinquencia*  *delinquencia femenina delinquencia juvenil*  *delincuencial*  *delincuenciales* |  |
| --- | --- | --- |

| **Database** | **Search strategy** | **Hits** |
| --- | --- | --- |
| **PsycINFO (Ovid)** 1967 to 2013 | 1. developing countries/ 2. (Africa or "Latin America" or Caribbean or "West Indies" or "Eastern Europe" or Soviet or "South America" or "Middle East" or "Latin America" or "Central America").hw,ti,ab. 3. (Afghanistan or Albania or Algeria or Angola or Antigua or Barbuda or Argentina or Armenia or Armenian or Aruba or Azerbaijan or Bahrain or Bangladesh or Barbados or Benin or Byelarus or Byelorussian or Belarus or Belorussian or Belorussia or Belize or Bhutan or Bolivia or Bosnia or Herzegovina or Hercegovina or Botswana or Brasil or Brazil or Bulgaria or Burkina Faso or Burkina Fasso or Upper Volta or Burundi or Urundi or Cambodia or Khmer Republic or Kampuchea or Cameroon or Cameroons or Cameron or Camerons or Cape Verde or Central African Republic or Chad or Chile or China or Colombia or Comoros or Comoro Islands or Comores or Mayotte or Congo or Zaire or Costa Rica or Cote d'Ivoire or Ivory Coast or Croatia or Cuba or Cyprus or Czechoslovakia or Czech Republic or Slovakia or Slovak Republic or Djibouti or French Somaliland or Dominica or Dominican Republic or East Timor or East Timur or Timor Leste or Ecuador or Egypt or United Arab Republic or El Salvador or Eritrea or Estonia or Ethiopia or Fiji or Gabon or Gabonese Republic or Gambia or Gaza or Georgia Republic or Georgian Republic or Ghana or Gold Coast or Greece or Grenada or Guatemala or Guinea or Guam or Guiana or Guyana or Haiti or Honduras or Hungary or India or Maldives or Indonesia or Iran or Iraq or Isle of Man or Jamaica or Jordan or Kazakhstan or Kazakh or Kenya or Kiribati or Korea or Kosovo or Kyrgyzstan or Kirghizia or Kyrgyz Republic or Kirghiz or Kirgizstan or Lao PDR or Laos or Latvia or Lebanon or Lesotho or Basutoland or Liberia or Libya or Lithuania or Macedonia or Madagascar or Malagasy Republic or Malaysia or Malaya or Malay or Sabah or Sarawak or Malawi or Nyasaland or Mali or Malta or Marshall Islands or Mauritania or Mauritius or Agalega Islands or Mexico or Micronesia or Middle East or Moldova or Moldovia or Moldovian or Mongolia or Montenegro or Morocco or Ifni or Mozambique or Myanmar or Myanma or Burma or Namibia or Nepal or Netherlands Antilles or New Caledonia or Nicaragua or Niger or Nigeria or Northern Mariana Islands or Oman or Muscat or Pakistan or Palau or Palestine or Panama or Paraguay or Peru or Philippines or Philipines or Phillipines or Phillippines or Poland or Portugal or Puerto Rico or Romania or Rumania or Roumania or Russia or Russian or Rwanda or Ruanda or Saint Kitts or St Kitts or Nevis or Saint Lucia or St Lucia or Saint Vincent or St Vincent or Grenadines or Samoa or Samoan Islands or Navigator Island or Navigator Islands or Sao Tome or Saudi Arabia or Senegal or Serbia or Montenegro or Seychelles or Sierra Leone or Slovenia or Sri Lanka or Ceylon or Solomon Islands or Somalia or South Africa or Sudan or Suriname or Surinam or Swaziland or Syria or Tajikistan or Tadzhikistan or Tadjikistan or Tadzhik or Tanzania or Thailand or Togo or Togolese Republic or Tonga or Trinidad or Tobago or Tunisia or Turkey or Turkmenistan or Turkmen or Uganda or Ukraine or Uruguay or USSR or Soviet Union or Union of Soviet Socialist Republics or Uzbekistan or Uzbek or Vanuatu or New Hebrides or Venezuela or Vietnam or Viet Nam or West Bank or Yemen or Yugoslavia or Zambia or Zimbabwe or Rhodesia).hw,ti,ab,cp. 4. ((developing or less* developed or under developed or underdeveloped or middle income or low* income or underserved or under served or deprived or poor* or foreign) adj (countr* or nation? or population? or world or region*)).hw,ti,ab. 5. ((developing or less* developed or under de veloped or underdeveloped or middle income or low* income) adj (economy or economies)).hw,ti,ab. 6. (lmic or lmics or third world or lami countr*).hw,ti,ab. 7. transitional countr*.hw,ti,ab. 8. OR/1-7 9. antisocial behavior/ 10. conduct disorder/ 11. exp behavior problems/ 12. behavior disorders/ 13. impulse control disorders/ 14. adjustment disorders/ 15. violence/ 16. exp violent crime/ 17. workplace violence/ 18. crime/ 19. criminal behavior/ 20. crime.mp. 21. crimes.mp. 22. criminal*.mp. 23. exp homicide/ 24. homicid*.mp. 25. exp perpetrators/ 26. attack behavior/ 27. acting out/ 28. exp gangs/ 29. gang.mp. 30. gangs.mp. 31. exp bullying/ 32. bully*.mp. 33. aggress*.mp. 34. aggressive behavior/ 35. (conduct adj1 problem*).mp. 36. (behavio?r adj1 problem*).mp. 37. (conduct adj1 disorder*).mp. 38. (behavio?r adj1 disorder*).mp. 39. (antisocial adj1 behavio?r*).mp. 40. (anti-social adj1 behavio?r*).mp. 41. (oppositional adj1 defiant adj1 disorder*).af. 42. (disruptive adj1 behavio?r adj1 disorder*).af. 43. (externalizing adj1 behavio?r adj1 problem*).mp. 44. externalizing.mp. 45. externalising.mp. 46. externalized.mp. 47. externalised.mp. 48. externaliz*.mp. 49. externalis*.mp. 50. (childhood adj1 externalizing adj1 behavio?r).mp. 51. (externalizing adj1 behavio?r).mp. 52. (externalising adj1 behavio?r).mp. 53. OR/9-52 54. exp [Childhood Development](http://ovidsp.uk.ovid.com/sp-3.9.1a/ovidweb.cgi?S=MMCLPDKNBGHFBPOIFNNKFCOFPKALAA00&Controlled+Vocabulary=thes+Childhood+Development&)/ 55. Adolescent development/ 56. Child Welfare/ 57. Child Care/ 58. baby.ti,ab. 59. babies.ti,ab. 60. toddler.ti,ab. 61. toddlers.ti,ab. 62. adolescen*.ti,ab. 63. adolescent.ti,ab. 64. adolescents.ti,ab. 65. adolescence.ti,ab. 66. child*.ti,ab. 67. child.ti,ab. 68. children*.ti,ab. 69. childhood*.ti,ab. 70. childhood.ti,ab. 71. youth*.ti,ab. 72. youth.ti,ab. 73. youths.ti,ab. 74. student*.ti,ab. 75. Students.ti,ab. 76. Student.ti,ab. 77. teen*.ti,ab. 78. teenager.ti,ab. 79. teenagers.ti,ab. 80. boy.ti,ab. 81. boys.ti,ab. 82. girl.ti,ab. 83. girls.ti,ab. 84. pupil.ti,ab. 85. pupils.ti,ab. 86. pupil*.ti,ab. 87. youngster*.ti,ab. 88. youngster.ti,ab. 89. youngsters.ti,ab. 90. juvenile*.ti,ab. 91. juvenile.ti,ab. 92. juveniles.ti,ab. 93. Infant*.ti,ab. 94. infant.ti,ab. 95. infants.ti,ab. 96. young adj1 adult*.ti,ab. 97. OR/54-96 98. 8 and 53 99. 97 and 98 100. exp juvenile delinquency/ 101. (juvenile adj1 delinquen*).mp. 102. school violence/ 103. OR/100-102 104. 8 and 103 | 4480 |
| **Ovid MEDLINE(R) In-Process & Other Non-Indexed Citations and Ovid MEDLINE(R) 1946 to Present** Ovid MEDLINE(R) In-Process & Other Non-Indexed Citations and Ovid MEDLINE(R) 1946 to Presen • Ovid MEDLINE(R) In-Process & Other Non-Indexed Citations and Ovid MEDLINE(R) 1946 to Prese | 1. Developing Countries.sh. 2. (Africa or Central Africa or Latin America or Caribbean or West Indies or Eastern Europe or Soviet or South America or Arab or Middle East or Latin America or Central America).hw,kf,ti,ab,cp. 3. (Afghanistan or Albania or Algeria or Angola or Antigua or Barbuda or Argentina or Armenia or Armenian or Aruba or Azerbaijan or Bahrain or Bangladesh or Barbados or Benin or Byelarus or Byelorussian or Belarus or Belorussian or Belorussia or Belize or Bhutan or Bolivia or Bosnia or Herzegovina or Hercegovina or Botswana or Brasil or Brazil or Bulgaria or Burkina Faso or Burkina Fasso or Upper Volta or Burundi or Urundi or Cambodia or Khmer Republic or Kampuchea or Cameroon or Cameroons or Cameron or Camerons or Cape Verde or Central African Republic or Chad or Chile or China or Colombia or Comoros or Comoro Islands or Comores or Mayotte or Congo or Zaire or Costa Rica or Cote d'Ivoire or Ivory Coast or Croatia or Cuba or Cyprus or Czechoslovakia or Czech Republic or Slovakia or Slovak Republic or Djibouti or French Somaliland or Dominica or Dominican Republic or East Timor or East Timur or Timor Leste or Ecuador or Egypt or United Arab Republic or El Salvador or Eritrea or Estonia or Ethiopia or Fiji or Gabon or Gabonese Republic or Gambia or Gaza or Georgia Republic or Georgian Republic or Ghana or Gold Coast or Greece or Grenada or Guatemala or Guinea or Guam or Guiana or Guyana or Haiti or Honduras or Hungary or India or Maldives or Indonesia or Iran or Iraq or Isle of Man or Jamaica or Jordan or Kazakhstan or Kazakh or Kenya or Kiribati or Korea or Kosovo or Kyrgyzstan or Kirghizia or Kyrgyz Republic or Kirghiz or Kirgizstan or Lao PDR or Laos or Latvia or Lebanon or Lesotho or Basutoland or Liberia or Libya or Lithuania or Macedonia or Madagascar or Malagasy Republic or Malaysia or Malaya or Malay or Sabah or Sarawak or Malawi or Nyasaland or Mali or Malta or Marshall Islands or Mauritania or Mauritius or Agalega Islands or Mexico or Micronesia or Middle East or Moldova or Moldovia or Moldovian or Mongolia or Montenegro or Morocco or Ifni or Mozambique or Myanmar or Myanma or Burma or Namibia or Nepal or Netherlands Antilles or New Caledonia or Nicaragua or Niger or Nigeria or Northern Mariana Islands or Oman or Muscat or Pakistan or Palau or Palestine or Panama or Paraguay or Peru or Philippines or Philipines or Phillipines or Phillippines or Poland or Portugal or Puerto Rico or Romania or Rumania or Roumania or Russia or Russian or Rwanda or Ruanda or Saint Kitts or St Kitts or Nevis or Saint Lucia or St Lucia or Saint Vincent or St Vincent or Grenadines or Samoa or Samoan Islands or Navigator Island or Navigator Islands or Sao Tome or Saudi Arabia or Senegal or Serbia or Montenegro or Seychelles or Sierra Leone or Slovenia or Sri Lanka or Ceylon or Solomon Islands or Somalia or South Africa or Sudan or Suriname or Surinam or Swaziland or Syria or Tajikistan or Tadzhikistan or Tadjikistan or Tadzhik or Tanzania or Thailand or Togo or Togolese Republic or Tonga or Trinidad or Tobago or Tunisia or Turkey or Turkmenistan or Turkmen or Uganda or Ukraine or Uruguay or USSR or Soviet Union or Union of Soviet Socialist Republics or Uzbekistan or Uzbek or Vanuatu or New Hebrides or Venezuela or Vietnam or Viet Nam or West Bank or Yemen or Yugoslavia or Zambia or Zimbabwe or Rhodesia).hw,kf,ti,ab,cp. 4. ((developing or less* developed or under developed or underdeveloped or middle income or low* income or underserved or under served or deprived or poor* or foreign) adj (countr* or nation? or population? or world or region*)).ti,ab. 5. ((developing or less* developed or under developed or underdeveloped or middle income or low* income) adj (economy or economies)).ti,ab. 6. (lmic or lmics or third world or lami countr*).ti,ab. 7. transitional countr*.ti,ab. 8. or/1-8 9. juvenile delinquency.sh. 10. (juvenile adj1 delinquen*).mp. 11. "Child Behavior Disorders".sh. 12. (school adj1 violence).mp. 13. (childhood adj1 externalizing adj1 behavio?r).mp.  or/9-13  1. 8 and 14 2. Social Behavior Disorders.sh. 3. conduct disorder.sh. 4. (conduct adj1 disorder*).mp. 5. aggression.sh. 6. aggress*.mp. 7. (acting adj1 out).mp. 8. (aggressive adj1 behavio?r).mp. 9. (behavio?r* adj1 problem*).mp. 10. (behavio?r* adj1 disorder*).mp. 11. (conduct adj1 problem*).mp. 12. (conduct adj1 disorder*).mp. 13. (impulse adj1 control adj1 disorder*).mp. 14. (antisocial adj1 behavio?r*).mp. 15. (anti-social adj1 behavio?r*).mp. 16. (oppositional adj1 defiant adj1 disorder*).af. 17. (disruptive adj1 behavio?r adj1 disorder*).af. 18. violen*.mp. 19. (violent adj1 crime*).mp. 20. exp crime/ 21. crime.mp. 22. crimes.mp. 23. criminal*.mp. 24. (criminal behavio?r*).mp. 25. bully*.mp 26. bullying.sh. 27. gang.mp. 28. gangs.mp. 29. homicid*.mp. 30. homicide.sh. 31. (externalizing adj1 behavio?r adj1 problem*).mp. 32. externalizing.mp. 33. externalising.mp. 34. externalized.mp. 35. externalised.mp. 36. externaliz*.mp. 37. externalis*.mp. 38. (externalizing adj1 behavio?r).mp. 39. or/16-52 40. exp child/ 41. "Child Health Services".sh. 42. "Child Welfare".sh. 43. "Child Behavior".sh. 44. "Child Care".sh. 45. “Child Development".sh. 46. Infant.sh. 47. baby.ti,ab. 48. babies.ti,ab. 49. toddler.ti,ab. 50. toddlers.ti,ab. 51. adolescen*.ti,ab. 52. adolescent.ti,ab. 53. adolescents.ti,ab. 54. adolescence.ti,ab. 55. child*.ti,ab. 56. child.ti,ab. 57. children*.ti,ab. 58. childhood*.ti,ab. 59. childhood.ti,ab. 60. youth*.ti,ab. 61. youth.ti,ab. 62. youths.ti,ab. 63. student*.ti,ab. 64. student.ti,ab. 65. students.ti,ab. 66. teen*.ti,ab. 67. teenager.ti,ab. 68. teenagers.ti,ab. 69. boy.ti,ab. 70. boys.ti,ab. 71. girl.ti,ab. 72. girls.ti,ab. 73. pupil.ti,ab. 74. pupils.ti,ab. 75. pupil*.ti,ab. 76. youngster*.ti,ab. 77. youngster.ti,ab. 78. youngsters.ti,ab. 79. juvenile*.ti,ab. 80. juvenile.ti,ab. 81. juveniles.ti,ab. 82. infant*.ti,ab. 83. infant.ti,ab. 84. infants.ti,ab. 85. (young adj1 adult*).ti,ab. 86. or/54-99 87. 8 and 53 and 100 | 11842 |
| **EMBASE (Ovid)**  1974 to 2013  Using EMTREE | 1. Exp developing country/ 2. (Developing adj1 Countr*).hw,ti,ab,cp. 3. (Africa or Central Africa or Latin America or Caribbean or West Indies or Eastern Europe or Soviet or South America or Arab or Middle East or Latin America or Central America).hw,ti,ab,cp. 4. (Afghanistan or Albania or Algeria or Angola or Antigua or Barbuda or Argentina or Armenia or Armenian or Aruba or Azerbaijan or Bahrain or Bangladesh or Barbados or Benin or Byelarus or Byelorussian or Belarus or Belorussian or Belorussia or Belize or Bhutan or Bolivia or Bosnia or Herzegovina or Hercegovina or Botswana or Brasil or Brazil or Bulgaria or Burkina Faso or Burkina Fasso or Upper Volta or Burundi or Urundi or Cambodia or Khmer Republic or Kampuchea or Cameroon or Cameroons or Cameron or Camerons or Cape Verde or Central African Republic or Chad or Chile or China or Colombia or Comoros or Comoro Islands or Comores or Mayotte or Congo or Zaire or Costa Rica or Cote d'Ivoire or Ivory Coast or Croatia or Cuba or Cyprus or Czechoslovakia or Czech Republic or Slovakia or Slovak Republic or Djibouti or French Somaliland or Dominica or Dominican Republic or East Timor or East Timur or Timor Leste or Ecuador or Egypt or United Arab Republic or El Salvador or Eritrea or Estonia or Ethiopia or Fiji or Gabon or Gabonese Republic or Gambia or Gaza or Georgia Republic or Georgian Republic or Ghana or Gold Coast or Greece or Grenada or Guatemala or Guinea or Guam or Guiana or Guyana or Haiti or Honduras or Hungary or India or Maldives or Indonesia or Iran or Iraq or Isle of Man or Jamaica or Jordan or Kazakhstan or Kazakh or Kenya or Kiribati or Korea or Kosovo or Kyrgyzstan or Kirghizia or Kyrgyz Republic or Kirghiz or Kirgizstan or Lao PDR or Laos or Latvia or Lebanon or Lesotho or Basutoland or Liberia or Libya or Lithuania or Macedonia or Madagascar or Malagasy Republic or Malaysia or Malaya or Malay or Sabah or Sarawak or Malawi or Nyasaland or Mali or Malta or Marshall Islands or Mauritania or Mauritius or Agalega Islands or Mexico or Micronesia or Middle East or Moldova or Moldovia or Moldovian or Mongolia or Montenegro or Morocco or Ifni or Mozambique or Myanmar or Myanma or Burma or Namibia or Nepal or Netherlands Antilles or New Caledonia or Nicaragua or Niger or Nigeria or Northern Mariana Islands or Oman or Muscat or Pakistan or Palau or Palestine or Panama or Paraguay or Peru or Philippines or Philipines or Phillipines or Phillippines or Poland or Portugal or Puerto Rico or Romania or Rumania or Roumania or Russia or Russian or Rwanda or Ruanda or Saint Kitts or St Kitts or Nevis or Saint Lucia or St Lucia or Saint Vincent or St Vincent or Grenadines or Samoa or Samoan Islands or Navigator Island or Navigator Islands or Sao Tome or Saudi Arabia or Senegal or Serbia or Montenegro or Seychelles or Sierra Leone or Slovenia or Sri Lanka or Ceylon or Solomon Islands or Somalia or South Africa or Sudan or Suriname or Surinam or Swaziland or Syria or Tajikistan or Tadzhikistan or Tadjikistan or Tadzhik or Tanzania or Thailand or Togo or Togolese Republic or Tonga or Trinidad or Tobago or Tunisia or Turkey or Turkmenistan or Turkmen or Uganda or Ukraine or Uruguay or USSR or Soviet Union or Union of Soviet Socialist Republics or Uzbekistan or Uzbek or Vanuatu or New Hebrides or Venezuela or Vietnam or Viet Nam or West Bank or Yemen or Yugoslavia or Zambia or Zimbabwe or Rhodesia).hw,ti,ab,cp. 5. ((developing or less* developed or under developed or underdeveloped or middle income or low* income or underserved or under served or deprived or poor* or foreign) adj1 (countr* or nation? or population? or world or region*)).ti,ab. 6. ((developing or less* developed or under developed or underdeveloped or middle income or low* income) adj1 (economy or economies)).ti,ab. 7. (low adj3 middle adj1 countr*).ti,ab. 8. (lmic or lmics or third world or lami countr*).ti,ab. 9. (transitional countr*).ti,ab. 10. or/1-9 11. exp delinquency/ 12. (juvenile adj1 delinquen*).mp. 13. (school adj1 violence).mp.  or/11-13  1. (conduct adj1 problem*).mp. 2. (conduct adj1 disorder*).mp. 3. (behavio?r* adj1 problem*).mp. 4. (behavio?r adj1 disorder*).mp. 5. (oppositional adj1 defiant adj1 disorder*).af. 6. (disruptive adj1 behavio?r adj1 disorder*).af. 7. (impulse adj1 control adj1 disorder*).mp. 8. (criminal adj1 behavio?r*).mp. 9. (violent adj1 crime*).mp. 10. homicid*.mp. 11. homicide.mp. 12. homicides.mp. 13. conduct disorder/ 14. aggression.mp. 15. aggressive.mp. 16. aggress*.mp. 17. violen*.mp. 18. violent.mp. 19. violence.mp. 20. crime.mp. 21. crimes.mp 22. criminal*.mp. 23. gang.mp. 24. gangs.mp. 25. bully*.mp. 26. bully.mp. 27. bullying.mp. 28. (aggressive adj1 behavio?r).mp. 29. (antisocial adj1 behavio?r).mp. 30. (anti-social adj1 behavio?r*).mp. 31. exp aggression/ 32. homicide/ 33. gang/ 34. crime/ 35. criminal behavior/ 36. abnormal behavior/ 37. behavior disorder/ 38. disruptive behaviour/ 39. criminology/ 40. homicide/ 41. acting out/ 42. violence/ 43. workplace violence/ 44. impulse control disorder/ 45. oppositional defiant disorder/ 46. conduct disorder/ 47. (externalizing adj1 behavio?r adj1 problem*).mp. 48. (externalizing adj1 behavio?r).mp. 49. (externalising adj1 behavio?r).mp. 50. externalizing.mp. 51. externalising.mp. 52. externalized.mp. 53. externalised.mp. 54. externaliz*.mp. 55. externalis*.mp. 56. or/15-69 57. exp child/ 58. adolescent.sh. 59. Infant.sh. 60. baby.ti,ab. 61. babies.ti,ab. 62. toddler.ti,ab. 63. toddlers.ti,ab. 64. adolescen*.ti,ab. 65. adolescent.ti,ab. 66. adolescents.ti,ab. 67. adolescence.ti,ab. 68. child*.ti,ab. 69. child.ti,ab. 70. children*.ti,ab. 71. childhood*.ti,ab. 72. childhood.ti,ab. 73. youth*.ti,ab. 74. youth.ti,ab. 75. youths.ti,ab. 76. student*.ti,ab. 77. students.ti,ab. 78. student.ti,ab. 79. teen*.ti,ab. 80. teenager.ti,ab. 81. teenagers.ti,ab. 82. boy.ti,ab. 83. boys.ti,ab. 84. girl.ti,ab. 85. girls.ti,ab. 86. pupil.ti,ab. 87. pupils.ti,ab. 88. pupil*.ti,ab. 89. youngster*.ti,ab. 90. youngster.ti,ab. 91. youngsters.ti,ab. 92. juvenile*.ti,ab. 93. juvenile.ti,ab. 94. juveniles.ti,ab. 95. Infant*.ti,ab. 96. infant.ti,ab. 97. infants.ti,ab. 98. (young adj1 adult*).ti,ab. 99. or/71-112 100. 10 and 70 101. 113 and 114 | 14260 |
| **CINAHL (EBSCO)** | 1. TI (“developing country” or “developing countries” or “developing nation” or “developing nations” or less* W1 “developed country” or less* W1 “developed countries” or less* W1 “developed nation” or less* W1 “developed nations” or “third world” or “under developed” or “middle income” or “low income” or “underserved country” or “underserved countries” or “underserved nation” or “underserved nations” or “under served country” or “under served countries” or “under served nation” or “under served nations” or “underserved population” or “underserved populations” or “under served population” or “under served populations” or “deprived country” or “deprived countries” or “deprived nation” or “deprived nations” or poor* W1 country or poor* W1 countries or poor* W1 nation* or poor* W1 population* or lmic or lmics) 2. AB (“developing country” or “developing countries” or “developing nation” or “developing nations” or less* W1 “developed country” or less* W1 “developed countries” or less* W1 “developed nation” or less* W1 “developed nations” or “third world” or “under developed” or “middle income” or “low income” or “underserved country” or “underserved countries” or “underserved nation” or “underserved nations” or “under served country” or “under served countries” or “under served nation” or “under served nations” or “underserved population” or “underserved populations” or “under served population” or “under served populations” or “deprived country” or “deprived countries” or “deprived nation” or “deprived nations” or poor* W1 country or poor* W1 countries or poor* W1 nation* or poor* W1 population* or lmic or lmics) 3. MW (Afghanistan or Bangladesh or Benin or “Burkina Faso” or Burundi or Cambodia or “Central African Republic” or Chad or Comoros or Congo or “Cote d’Ivoire” or Eritrea or Ethiopia or Gambia or Ghana or Guinea or Haiti or India or Kenya or Korea or Kyrgyz or Kyrgyzstan or Lao or Laos or Liberia or Madagascar or Malawi or Mali or Mauritania or Melanesia or Mongolia or Mozambique or Burma or Myanmar or Nepal or Niger or Nigeria or Pakistan or Rwanda or “Salomon Islands” or “Sao Tome” or Senegal or “Sierra Leone” or Somalia or Sudan or Tajikistan or Tanzania or Timor or Togo or Uganda or Uzbekistan or Vietnam or “Viet Nam” or Yemen or Zambia or Zimbabwe) 4. TI (Afghanistan or Bangladesh or Benin or “Burkina Faso” or Burundi or Cambodia or “Central African Republic” or Chad or Comoros or Congo or “Cote d’Ivoire” or Eritrea or Ethiopia or Gambia or Ghana or Guinea or Haiti or India or Kenya or Korea or Kyrgyz or Kyrgyzstan or Lao or Laos or Liberia or Madagascar or Malawi or Mali or Mauritania or Melanesia or Mongolia or Mozambique or Burma or Myanmar or Nepal or Niger or Nigeria or Pakistan or Rwanda or “Salomon Islands” or “Sao Tome” or Senegal or “Sierra Leone” or Somalia or Sudan or Tajikistan or Tanzania or Timor or Togo or Uganda or Uzbekistan or Vietnam or “Viet Nam” or Yemen or Zambia or Zimbabwe) 5. AB (Afghanistan or Bangladesh or Benin or “Burkina Faso” or Burundi or Cambodia or “Central African Republic” or Chad or Comoros or Congo or “Cote d’Ivoire” or Eritrea or Ethiopia or Gambia or Ghana or Guinea or Haiti or India or Kenya or Korea or Kyrgyz or Kyrgyzstan or Lao or Laos or Liberia or Madagascar or Malawi or Mali or Mauritania or Melanesia or Mongolia or Mozambique or Burma or Myanmar or Nepal or Niger or Nigeria or Pakistan or Rwanda or “Salomon Islands” or “Sao Tome” or Senegal or “Sierra Leone” or Somalia or Sudan or Tajikistan or Tanzania or Timor or Togo or Uganda or Uzbekistan or Vietnam or “Viet Nam” or Yemen or Zambia or Zimbabwe) 6. MW (Albania or Algeria or Angola or Armenia or Azerbaijan or Belarus or Bhutan or Bolivia or Bosnia or Herzegovina or “Cape Verde” or Cameroon or China or Colombia or Congo or Cuba or Djibouti or “Dominican Republic” or Ecuador or Egypt or “El Salvador” or Fiji or Gaza or Georgia or Guam or Guatemala or Guyana or Honduras or “Indian Ocean Islands” or Indonesia or Iran or Iraq or Jamaica or Jordan or Kiribati or Lesotho or Macedonia or Maldives or “Marshall Islands” or Micronesia or “Middle East” or Moldova or Morocco or Namibia or Nicaragua or Palestin* or Paraguay or Peru or Philippines or Samoa or “Sri Lanka” or Suriname or Swaziland or Syria or “Syrian Arab Republic” or Thailand or Tonga or Tunisia or Turkmenistan or Ukraine or Vanuatu or “West Bank” ) or TI ( Albania or Algeria or Angola or Armenia or Azerbaijan or Belarus or Bhutan or Bolivia or Bosnia or Herzegovina or “Cape Verde” or Cameroon or China or Colombia or Congo or Cuba or Djibouti or “Dominican Republic” or Ecuador or Egypt or “El Salvador” or Fiji or Gaza or Georgia or Guam or Guatemala or Guyana or Honduras or “Indian Ocean Islands” or Indonesia or Iran or Iraq or Jamaica or Jordan or Kiribati or Lesotho or Macedonia or Maldives or “Marshall Islands” or Micronesia or “Middle East” or Moldova or Morocco or Namibia or Nicaragua or Palestin* or Paraguay or Peru or Philippines or Samoa or “Sri Lanka” or Suriname or Swaziland or Syria or “Syrian Arab Republic” or Thailand or Tonga or Tunisia or Turkmenistan or Ukraine or Vanuatu or “West Bank” Albania or Algeria or Angola or Armenia or Azerbaijan or Belarus or Bhutan or Bolivia or Bosnia or Herzegovina or “Cape Verde” or Cameroon or China or Colombia or Congo or Cuba or Djibouti or “Dominican Republic” or Ecuador or Egypt or “El Salvador” or Fiji or Gaza or Georgia or Guam or Guatemala or Guyana or Honduras or “Indian Ocean Islands” or Indonesia or Iran or Iraq or Jamaica or Jordan or Kiribati or Lesotho or Macedonia or Maldives or “Marshall Islands” or Micronesia or “Middle East” or Moldova or Morocco or Namibia or Nicaragua or Palestin* or Paraguay or Peru or Philippines or Samoa or “Sri Lanka” or Suriname or Swaziland or Syria or “Syrian Arab Republic” or Thailand or Tonga or Tunisia or Turkmenistan or Ukraine or Vanuatu or “West Bank”) 7. AB (Albania or Algeria or Angola or Armenia or Azerbaijan or Belarus or Bhutan or Bolivia or Bosnia or Herzegovina or “Cape Verde” or Cameroon or China or Colombia or Congo or Cuba or Djibouti or “Dominican Republic” or Ecuador or Egypt or “El Salvador” or Fiji or Gaza or Georgia or Guam or Guatemala or Guyana or Honduras or “Indian Ocean Islands” or Indonesia or Iran or Iraq or Jamaica or Jordan or Kiribati or Lesotho or Macedonia or Maldives or “Marshall Islands” or Micronesia or “Middle East” or Moldova or Morocco or Namibia or Nicaragua or Palestin* or Paraguay or Peru or Philippines or Samoa or “Sri Lanka” or Suriname or Swaziland or Syria or “Syrian Arab Republic” or Thailand or Tonga or Tunisia or Turkmenistan or Ukraine or Vanuatu or “West Bank”) 8. MW (“American Samoa” or Argentina or Belize or Botswana or Brazil or Bulgaria or Chile or Comoros or “Costa Rica” or Croatia or Dominica or Guinea or Gabon or Grenada or Grenadines or Hungary or Kazakhstan or Latvia or Lebanon or Libia or libyan or Libya or Lithuania or Malaysia or Mauritius or Mayotte or Mexico or Micronesia or Montenegro or Nevis or “Northern Mariana Islands” or Oman or Palau or Panama or Poland or Romania or Russia or “Russian Federation” or Samoa or “Saint Lucia” or “St Lucia” or “Saint Kitts” or “St Kitts” or “Saint Vincent” or “St Vincent” or Serbia or Seychelles or Slovakia or “Slovak Republic” or “South Africa” or Turkey or Uruguay or Venezuela or Yugoslavia) 9. TI (“American Samoa” or Argentina or Belize or Botswana or Brazil or Bulgaria or Chile or Comoros or “Costa Rica” or Croatia or Dominica or Guinea or Gabon or Grenada or Grenadines or Hungary or Kazakhstan or Latvia or Lebanon or Libia or libyan or Libya or Lithuania or Malaysia or Mauritius or Mayotte or Mexico or Micronesia or Montenegro or Nevis or “Northern Mariana Islands” or Oman or Palau or Panama or Poland or Romania or Russia or “Russian Federation” or Samoa or “Saint Lucia” or “St Lucia” or “Saint Kitts” or “St Kitts” or “Saint Vincent” or “St Vincent” or Serbia or Seychelles or Slovakia or “Slovak Republic” or “South Africa” or Turkey or Uruguay or Venezuela or Yugoslavia) 10. AB (“American Samoa” or Argentina or Belize or Botswana or Brazil or Bulgaria or Chile or Comoros or “Costa Rica” or Croatia or Dominica or Guinea or Gabon or Grenada or Grenadines or Hungary or Kazakhstan or Latvia or Lebanon or Libia or libyan or Libya or Lithuania or Malaysia or Mauritius or Mayotte or Mexico or Micronesia or Montenegro or Nevis or “Northern Mariana Islands” or Oman or Palau or Panama or Poland or Romania or Russia or “Russian Federation” or Samoa or “Saint Lucia” or “St Lucia” or “Saint Kitts” or “St Kitts” or “Saint Vincent” or “St Vincent” or Serbia or Seychelles or Slovakia or “Slovak Republic” or “South Africa” or Turkey or Uruguay or Venezuela or Yugoslavia) 11. TI (Africa or Asia or “South America” or “Latin America” or “Central America”) 12. AB (Africa or Asia or “South America” or “Latin America” or “Central America”) 13. (MH “Asia+”) 14. (MH “West Indies+”) 15. (MH “South America+”) 16. (MH “Latin America”) 17. (MH “Central America+”) 18. (MH “Africa+”) 19. (MH “Developing Countries”)  or/1-19  1. (MH "Juvenile Delinquency") 2. AB (juvenile N1 delinquen*) 3. AB (school N1 violence) 4. (MH "Juvenile Offenders+") 5. (MH "[Child Behavior Disorders](http://web.ebscohost.com/ehost/mesh/tree?term=Child%20Behavior%20Disorders&sid=a3e3919d-8eb9-4b24-8097-5c21bd819813%40sessionmgr110&vid=15)")  or/21-2520 and 26  1. (MH "Aggression") 2. (MH "Social Behavior Disorders") 3. (MH "Crime") 4. (MH "Violence") 5. (MH "Homicide") 6. (MH "Assault and Battery") 7. (MH "Aggression+") 8. AB (conduct N1 problem*) 9. AB (behavio#r N1 problem*) 10. AB (antisocial N1 behavio#r) 11. AB (disruptive N1 behavio#r) 12. AB (conduct N1 disorder*) 13. AB (behavio#r N1 disorder*) 14. AB (aggressive N1 behavio#r) 15. AB (aggression) 16. AB (aggressive) 17. AB (antisocial N1 behavio#r) 18. AB (anti-social N1 behavio#r) 19. AB (gang) 20. AB (gangs) 21. AB (criminal N1 behavio#r) 22. AB (violent N1 crime) 23. AB (homicid*) 24. AB (violence) 25. AB (violent) 26. AB (crime) 27. AB (crimes) 28. AB (criminal*) 29. AB (bully) 30. AB (bullying) 31. AB (delinquent*)  \|  \| \| --- \|  1. AB (delinquenc*) 2. TX (oppositional N1 defiant N1 disorder*) 3. TX (disruptive N1 behavio#r N1 disorder*) 4. AB (externalizing N1 behavio#r N1 problem*) 5. AB (externalizing) 6. AB (externalising) 7. AB (externalized) 8. AB (externalised) 9. AB (externaliz*) 10. AB (externalis*) 11. AB (externalizing N1 behavio#r) 12. AB (externalising N1 behavio#r) 13. or /28-71 14. 20 AND 72 15. (MH " Child+") 16. (MH "Adolescence") 17. AB (Adolescen*) 18. AB (Adolescence) 19. AB (Adolescent) 20. AB (adolescents) 21. AB (Child*) 22. AB (child) 23. AB (children) 24. AB (childhood) 25. AB (youth*) 26. AB (youth) 27. AB (youths) 28. AB (student*) 29. AB (Students) 30. AB (Student) 31. AB (teen*) 32. AB (teenager) 33. AB (teenagers) 34. AB (boy*) 35. AB (boy) 36. AB (boys) 37. AB (girl*) 38. AB (girl) 39. AB (girls) 40. AB (pupil) 41. AB (pupils) 42. AB (pupil*) 43. AB (youngster*) 44. AB (youngster) 45. AB (youngsters) 46. AB (juvenile*) 47. AB (juvenile) 48. AB (juveniles) 49. AB (young N1 adult*) 50. AB (infant*) 51. AB (infants) 52. AB (infant) 53. AB (baby*) 54. AB (baby) 55. AB (babies) 56. AB (toddler) 57. AB (toddler*) 58. AB (toddlers) 59. or/74-117 60. 20 and 118 | 3052 |
| **Criminal Justice Abstracts (EBSCOHost)** | 1. TI (“developing country” or “developing countries” or “developing nation” or “developing nations” or less* W1 “developed country” or less* W1 “developed countries” or less* W1 “developed nation” or less* W1 “developed nations” or “third world” or “under developed” or “middle income” or “low income” or “underserved country” or “underserved countries” or “underserved nation” or “underserved nations” or “under served country” or “under served countries” or “under served nation” or “under served nations” or “underserved population” or “underserved populations” or “under served population” or “under served populations” or “deprived country” or “deprived countries” or “deprived nation” or “deprived nations” or poor* W1 country or poor* W1 countries or poor* W1 nation* or poor* W1 population* or lmic or lmics) 2. AB (“developing country” or “developing countries” or “developing nation” or “developing nations” or less* W1 “developed country” or less* W1 “developed countries” or less* W1 “developed nation” or less* W1 “developed nations” or “third world” or “under developed” or “middle income” or “low income” or “underserved country” or “underserved countries” or “underserved nation” or “underserved nations” or “under served country” or “under served countries” or “under served nation” or “under served nations” or “underserved population” or “underserved populations” or “under served population” or “under served populations” or “deprived country” or “deprived countries” or “deprived nation” or “deprived nations” or poor* W1 country or poor* W1 countries or poor* W1 nation* or poor* W1 population* or lmic or lmics) 3. MW (Afghanistan or Bangladesh or Benin or “Burkina Faso” or Burundi or Cambodia or “Central African Republic” or Chad or Comoros or Congo or “Cote d’Ivoire” or Eritrea or Ethiopia or Gambia or Ghana or Guinea or Haiti or India or Kenya or Korea or Kyrgyz or Kyrgyzstan or Lao or Laos or Liberia or Madagascar or Malawi or Mali or Mauritania or Melanesia or Mongolia or Mozambique or Burma or Myanmar or Nepal or Niger or Nigeria or Pakistan or Rwanda or “Salomon Islands” or “Sao Tome” or Senegal or “Sierra Leone” or Somalia or Sudan or Tajikistan or Tanzania or Timor or Togo or Uganda or Uzbekistan or Vietnam or “Viet Nam” or Yemen or Zambia or Zimbabwe) 4. TI (Afghanistan or Bangladesh or Benin or “Burkina Faso” or Burundi or Cambodia or “Central African Republic” or Chad or Comoros or Congo or “Cote d’Ivoire” or Eritrea or Ethiopia or Gambia or Ghana or Guinea or Haiti or India or Kenya or Korea or Kyrgyz or Kyrgyzstan or Lao or Laos or Liberia or Madagascar or Malawi or Mali or Mauritania or Melanesia or Mongolia or Mozambique or Burma or Myanmar or Nepal or Niger or Nigeria or Pakistan or Rwanda or “Salomon Islands” or “Sao Tome” or Senegal or “Sierra Leone” or Somalia or Sudan or Tajikistan or Tanzania or Timor or Togo or Uganda or Uzbekistan or Vietnam or “Viet Nam” or Yemen or Zambia or Zimbabwe) 5. AB (Afghanistan or Bangladesh or Benin or “Burkina Faso” or Burundi or Cambodia or “Central African Republic” or Chad or Comoros or Congo or “Cote d’Ivoire” or Eritrea or Ethiopia or Gambia or Ghana or Guinea or Haiti or India or Kenya or Korea or Kyrgyz or Kyrgyzstan or Lao or Laos or Liberia or Madagascar or Malawi or Mali or Mauritania or Melanesia or Mongolia or Mozambique or Burma or Myanmar or Nepal or Niger or Nigeria or Pakistan or Rwanda or “Salomon Islands” or “Sao Tome” or Senegal or “Sierra Leone” or Somalia or Sudan or Tajikistan or Tanzania or Timor or Togo or Uganda or Uzbekistan or Vietnam or “Viet Nam” or Yemen or Zambia or Zimbabwe) 6. MW (Albania or Algeria or Angola or Armenia or Azerbaijan or Belarus or Bhutan or Bolivia or Bosnia or Herzegovina or “Cape Verde” or Cameroon or China or Colombia or Congo or Cuba or Djibouti or “Dominican Republic” or Ecuador or Egypt or “El Salvador” or Fiji or Gaza or Georgia or Guam or Guatemala or Guyana or Honduras or “Indian Ocean Islands” or Indonesia or Iran or Iraq or Jamaica or Jordan or Kiribati or Lesotho or Macedonia or Maldives or “Marshall Islands” or Micronesia or “Middle East” or Moldova or Morocco or Namibia or Nicaragua or Palestin* or Paraguay or Peru or Philippines or Samoa or “Sri Lanka” or Suriname or Swaziland or Syria or “Syrian Arab Republic” or Thailand or Tonga or Tunisia or Turkmenistan or Ukraine or Vanuatu or “West Bank” ) or TI ( Albania or Algeria or Angola or Armenia or Azerbaijan or Belarus or Bhutan or Bolivia or Bosnia or Herzegovina or “Cape Verde” or Cameroon or China or Colombia or Congo or Cuba or Djibouti or “Dominican Republic” or Ecuador or Egypt or “El Salvador” or Fiji or Gaza or Georgia or Guam or Guatemala or Guyana or Honduras or “Indian Ocean Islands” or Indonesia or Iran or Iraq or Jamaica or Jordan or Kiribati or Lesotho or Macedonia or Maldives or “Marshall Islands” or Micronesia or “Middle East” or Moldova or Morocco or Namibia or Nicaragua or Palestin* or Paraguay or Peru or Philippines or Samoa or “Sri Lanka” or Suriname or Swaziland or Syria or “Syrian Arab Republic” or Thailand or Tonga or Tunisia or Turkmenistan or Ukraine or Vanuatu or “West Bank” Albania or Algeria or Angola or Armenia or Azerbaijan or Belarus or Bhutan or Bolivia or Bosnia or Herzegovina or “Cape Verde” or Cameroon or China or Colombia or Congo or Cuba or Djibouti or “Dominican Republic” or Ecuador or Egypt or “El Salvador” or Fiji or Gaza or Georgia or Guam or Guatemala or Guyana or Honduras or “Indian Ocean Islands” or Indonesia or Iran or Iraq or Jamaica or Jordan or Kiribati or Lesotho or Macedonia or Maldives or “Marshall Islands” or Micronesia or “Middle East” or Moldova or Morocco or Namibia or Nicaragua or Palestin* or Paraguay or Peru or Philippines or Samoa or “Sri Lanka” or Suriname or Swaziland or Syria or “Syrian Arab Republic” or Thailand or Tonga or Tunisia or Turkmenistan or Ukraine or Vanuatu or “West Bank”) 7. AB (Albania or Algeria or Angola or Armenia or Azerbaijan or Belarus or Bhutan or Bolivia or Bosnia or Herzegovina or “Cape Verde” or Cameroon or China or Colombia or Congo or Cuba or Djibouti or “Dominican Republic” or Ecuador or Egypt or “El Salvador” or Fiji or Gaza or Georgia or Guam or Guatemala or Guyana or Honduras or “Indian Ocean Islands” or Indonesia or Iran or Iraq or Jamaica or Jordan or Kiribati or Lesotho or Macedonia or Maldives or “Marshall Islands” or Micronesia or “Middle East” or Moldova or Morocco or Namibia or Nicaragua or Palestin* or Paraguay or Peru or Philippines or Samoa or “Sri Lanka” or Suriname or Swaziland or Syria or “Syrian Arab Republic” or Thailand or Tonga or Tunisia or Turkmenistan or Ukraine or Vanuatu or “West Bank”) 8. MW (“American Samoa” or Argentina or Belize or Botswana or Brazil or Bulgaria or Chile or Comoros or “Costa Rica” or Croatia or Dominica or Guinea or Gabon or Grenada or Grenadines or Hungary or Kazakhstan or Latvia or Lebanon or Libia or libyan or Libya or Lithuania or Malaysia or Mauritius or Mayotte or Mexico or Micronesia or Montenegro or Nevis or “Northern Mariana Islands” or Oman or Palau or Panama or Poland or Romania or Russia or “Russian Federation” or Samoa or “Saint Lucia” or “St Lucia” or “Saint Kitts” or “St Kitts” or “Saint Vincent” or “St Vincent” or Serbia or Seychelles or Slovakia or “Slovak Republic” or “South Africa” or Turkey or Uruguay or Venezuela or Yugoslavia) 9. TI (“American Samoa” or Argentina or Belize or Botswana or Brazil or Bulgaria or Chile or Comoros or “Costa Rica” or Croatia or Dominica or Guinea or Gabon or Grenada or Grenadines or Hungary or Kazakhstan or Latvia or Lebanon or Libia or libyan or Libya or Lithuania or Malaysia or Mauritius or Mayotte or Mexico or Micronesia or Montenegro or Nevis or “Northern Mariana Islands” or Oman or Palau or Panama or Poland or Romania or Russia or “Russian Federation” or Samoa or “Saint Lucia” or “St Lucia” or “Saint Kitts” or “St Kitts” or “Saint Vincent” or “St Vincent” or Serbia or Seychelles or Slovakia or “Slovak Republic” or “South Africa” or Turkey or Uruguay or Venezuela or Yugoslavia) 10. AB (“American Samoa” or Argentina or Belize or Botswana or Brazil or Bulgaria or Chile or Comoros or “Costa Rica” or Croatia or Dominica or Guinea or Gabon or Grenada or Grenadines or Hungary or Kazakhstan or Latvia or Lebanon or Libia or libyan or Libya or Lithuania or Malaysia or Mauritius or Mayotte or Mexico or Micronesia or Montenegro or Nevis or “Northern Mariana Islands” or Oman or Palau or Panama or Poland or Romania or Russia or “Russian Federation” or Samoa or “Saint Lucia” or “St Lucia” or “Saint Kitts” or “St Kitts” or “Saint Vincent” or “St Vincent” or Serbia or Seychelles or Slovakia or “Slovak Republic” or “South Africa” or Turkey or Uruguay or Venezuela or Yugoslavia) 11. TI (Africa or Asia or “South America” or “Latin America” or “Central America”) 12. AB (Africa or Asia or “South America” or “Latin America” or “Central America”) 13. (MH “Asia+”) 14. (MH “West Indies+”) 15. (MH “South America+”) 16. (MH “Latin America”) 17. (MH “Central America+”) 18. (MH “Africa+”) 19. (MH “Developing Countries”)  or/1-19  1. (MH "Juvenile Delinquency") 2. AB (juvenile N1 delinquen*) 3. AB (school N1 violence) 4. (MH "Juvenile Offenders+") 5. (MH "[Child Behavior Disorders](http://web.ebscohost.com/ehost/mesh/tree?term=Child%20Behavior%20Disorders&sid=a3e3919d-8eb9-4b24-8097-5c21bd819813%40sessionmgr110&vid=15)")  or/21-2520 AND 26  1. (MH "Aggression") 2. (MH "Social Behavior Disorders") 3. (MH "Crime") 4. (MH "Violence") 5. (MH "Homicide") 6. (MH "Assault and Battery") 7. (MH "Aggression+") 8. AB (conduct N1 problem*) 9. AB (behavio#r N1 problem*) 10. AB (disruptive N1 behavio#r) 11. AB (conduct N1 disorder*) 12. AB (behavio#r N1 disorder*) 13. AB (aggressive N1 behavio#r) 14. AB (aggression) 15. AB (aggressive) 16. AB (antisocial N1 behavio#r) 17. AB (anti-social N1 behavio#r) 18. AB (gang) 19. AB (gangs) 20. AB (criminal N1 behavio#r) 21. AB (violent N1 crime) 22. AB (homicid*) 23. AB (violence) 24. AB (violent) 25. AB (crime) 26. AB (crimes) 27. AB (criminal*) 28. AB (bully) 29. AB (bullying) 30. AB (delinquent*)  \|  \| \| --- \|  1. AB (delinquenc*) 2. TX (oppositional N1 defiant N1 disorder*) 3. TX (disruptive N1 behavio#r N1 disorder*) 4. AB (externalizing N1 behavio#r N1 problem*) 5. AB (externalizing) 6. AB (externalising) 7. AB (externalized) 8. AB (externalised) 9. AB (externaliz*) 10. AB (externalis*) 11. AB (externalizing N1 behavio#r) 12. AB (externalising N1 behavio#r) 13. or /28-70 14. 20 AND 71 15. (MH " Child+") 16. (MH "Adolescence") 17. AB (Adolescen*) 18. AB (Adolescence) 19. AB (Adolescent) 20. AB (adolescents) 21. AB (Child*) 22. AB (child) 23. AB (children) 24. AB (childhood) 25. AB (youth*) 26. AB (youth) 27. AB (youths) 28. AB (student*) 29. AB (Students) 30. AB (Student) 31. AB (teen*) 32. AB (teenager) 33. AB (teenagers) 34. AB (boy*) 35. AB (boy) 36. AB (boys) 37. AB (girl*) 38. AB (girl) 39. AB (girls) 40. AB (pupil) 41. AB (pupils) 42. AB (pupil*) 43. AB (youngster*) 44. AB (youngster) 45. AB (youngsters) 46. AB (juvenile*) 47. AB (juvenile) 48. AB (juveniles) 49. AB (young N1 adult*) 50. AB (infant*) 51. AB (infants) 52. AB (infant) 53. AB (baby*) 54. AB (baby) 55. AB (babies) 56. AB (toddler) 57. AB (toddler*) 58. AB (toddlers) 59. or/73-116 60. 20 and 117 | 4,168 |
| [**Russian Academy of Sciences Bibliographies**](http://search.ebscohost.com/login.aspx?authtype=ip,shib&profile=ehost&defaultdb=rsb&custid=s3859159) (EBSCOHost) | Same as EconLit | 68 |
| **EconLit (EBSCOhost)** | 1. TI (“developing country” or “developing countries” or “developing nation” or “developing nations” or less* W1 “developed country” or less* W1 “developed countries” or less* W1 “developed nation” or less* W1 “developed nations” or “third world” or “under developed” or “middle income” or “low income” or “underserved country” or “underserved countries” or “underserved nation” or “underserved nations” or “under served country” or “under served countries” or “under served nation” or “under served nations” or “underserved population” or “underserved populations” or “under served population” or “under served populations” or “deprived country” or “deprived countries” or “deprived nation” or “deprived nations” or poor* W1 country or poor* W1 countries or poor* W1 nation* or poor* W1 population* or lmic or lmics) 2. AB (“developing country” or “developing countries” or “developing nation” or “developing nations” or less* W1 “developed country” or less* W1 “developed countries” or less* W1 “developed nation” or less* W1 “developed nations” or “third world” or “under developed” or “middle income” or “low income” or “underserved country” or “underserved countries” or “underserved nation” or “underserved nations” or “under served country” or “under served countries” or “under served nation” or “under served nations” or “underserved population” or “underserved populations” or “under served population” or “under served populations” or “deprived country” or “deprived countries” or “deprived nation” or “deprived nations” or poor* W1 country or poor* W1 countries or poor* W1 nation* or poor* W1 population* or lmic or lmics) 3. MW (Afghanistan or Bangladesh or Benin or “Burkina Faso” or Burundi or Cambodia or “Central African Republic” or Chad or Comoros or Congo or “Cote d’Ivoire” or Eritrea or Ethiopia or Gambia or Ghana or Guinea or Haiti or India or Kenya or Korea or Kyrgyz or Kyrgyzstan or Lao or Laos or Liberia or Madagascar or Malawi or Mali or Mauritania or Melanesia or Mongolia or Mozambique or Burma or Myanmar or Nepal or Niger or Nigeria or Pakistan or Rwanda or “Salomon Islands” or “Sao Tome” or Senegal or “Sierra Leone” or Somalia or Sudan or Tajikistan or Tanzania or Timor or Togo or Uganda or Uzbekistan or Vietnam or “Viet Nam” or Yemen or Zambia or Zimbabwe) 4. TI (Afghanistan or Bangladesh or Benin or “Burkina Faso” or Burundi or Cambodia or “Central African Republic” or Chad or Comoros or Congo or “Cote d’Ivoire” or Eritrea or Ethiopia or Gambia or Ghana or Guinea or Haiti or India or Kenya or Korea or Kyrgyz or Kyrgyzstan or Lao or Laos or Liberia or Madagascar or Malawi or Mali or Mauritania or Melanesia or Mongolia or Mozambique or Burma or Myanmar or Nepal or Niger or Nigeria or Pakistan or Rwanda or “Salomon Islands” or “Sao Tome” or Senegal or “Sierra Leone” or Somalia or Sudan or Tajikistan or Tanzania or Timor or Togo or Uganda or Uzbekistan or Vietnam or “Viet Nam” or Yemen or Zambia or Zimbabwe) 5. AB (Afghanistan or Bangladesh or Benin or “Burkina Faso” or Burundi or Cambodia or “Central African Republic” or Chad or Comoros or Congo or “Cote d’Ivoire” or Eritrea or Ethiopia or Gambia or Ghana or Guinea or Haiti or India or Kenya or Korea or Kyrgyz or Kyrgyzstan or Lao or Laos or Liberia or Madagascar or Malawi or Mali or Mauritania or Melanesia or Mongolia or Mozambique or Burma or Myanmar or Nepal or Niger or Nigeria or Pakistan or Rwanda or “Salomon Islands” or “Sao Tome” or Senegal or “Sierra Leone” or Somalia or Sudan or Tajikistan or Tanzania or Timor or Togo or Uganda or Uzbekistan or Vietnam or “Viet Nam” or Yemen or Zambia or Zimbabwe) 6. MW (Albania or Algeria or Angola or Armenia or Azerbaijan or Belarus or Bhutan or Bolivia or Bosnia or Herzegovina or “Cape Verde” or Cameroon or China or Colombia or Congo or Cuba or Djibouti or “Dominican Republic” or Ecuador or Egypt or “El Salvador” or Fiji or Gaza or Georgia or Guam or Guatemala or Guyana or Honduras or “Indian Ocean Islands” or Indonesia or Iran or Iraq or Jamaica or Jordan or Kiribati or Lesotho or Macedonia or Maldives or “Marshall Islands” or Micronesia or “Middle East” or Moldova or Morocco or Namibia or Nicaragua or Palestin* or Paraguay or Peru or Philippines or Samoa or “Sri Lanka” or Suriname or Swaziland or Syria or “Syrian Arab Republic” or Thailand or Tonga or Tunisia or Turkmenistan or Ukraine or Vanuatu or “West Bank” ) or TI ( Albania or Algeria or Angola or Armenia or Azerbaijan or Belarus or Bhutan or Bolivia or Bosnia or Herzegovina or “Cape Verde” or Cameroon or China or Colombia or Congo or Cuba or Djibouti or “Dominican Republic” or Ecuador or Egypt or “El Salvador” or Fiji or Gaza or Georgia or Guam or Guatemala or Guyana or Honduras or “Indian Ocean Islands” or Indonesia or Iran or Iraq or Jamaica or Jordan or Kiribati or Lesotho or Macedonia or Maldives or “Marshall Islands” or Micronesia or “Middle East” or Moldova or Morocco or Namibia or Nicaragua or Palestin* or Paraguay or Peru or Philippines or Samoa or “Sri Lanka” or Suriname or Swaziland or Syria or “Syrian Arab Republic” or Thailand or Tonga or Tunisia or Turkmenistan or Ukraine or Vanuatu or “West Bank” Albania or Algeria or Angola or Armenia or Azerbaijan or Belarus or Bhutan or Bolivia or Bosnia or Herzegovina or “Cape Verde” or Cameroon or China or Colombia or Congo or Cuba or Djibouti or “Dominican Republic” or Ecuador or Egypt or “El Salvador” or Fiji or Gaza or Georgia or Guam or Guatemala or Guyana or Honduras or “Indian Ocean Islands” or Indonesia or Iran or Iraq or Jamaica or Jordan or Kiribati or Lesotho or Macedonia or Maldives or “Marshall Islands” or Micronesia or “Middle East” or Moldova or Morocco or Namibia or Nicaragua or Palestin* or Paraguay or Peru or Philippines or Samoa or “Sri Lanka” or Suriname or Swaziland or Syria or “Syrian Arab Republic” or Thailand or Tonga or Tunisia or Turkmenistan or Ukraine or Vanuatu or “West Bank”) 7. AB (Albania or Algeria or Angola or Armenia or Azerbaijan or Belarus or Bhutan or Bolivia or Bosnia or Herzegovina or “Cape Verde” or Cameroon or China or Colombia or Congo or Cuba or Djibouti or “Dominican Republic” or Ecuador or Egypt or “El Salvador” or Fiji or Gaza or Georgia or Guam or Guatemala or Guyana or Honduras or “Indian Ocean Islands” or Indonesia or Iran or Iraq or Jamaica or Jordan or Kiribati or Lesotho or Macedonia or Maldives or “Marshall Islands” or Micronesia or “Middle East” or Moldova or Morocco or Namibia or Nicaragua or Palestin* or Paraguay or Peru or Philippines or Samoa or “Sri Lanka” or Suriname or Swaziland or Syria or “Syrian Arab Republic” or Thailand or Tonga or Tunisia or Turkmenistan or Ukraine or Vanuatu or “West Bank”) 8. MW (“American Samoa” or Argentina or Belize or Botswana or Brazil or Bulgaria or Chile or Comoros or “Costa Rica” or Croatia or Dominica or Guinea or Gabon or Grenada or Grenadines or Hungary or Kazakhstan or Latvia or Lebanon or Libia or libyan or Libya or Lithuania or Malaysia or Mauritius or Mayotte or Mexico or Micronesia or Montenegro or Nevis or “Northern Mariana Islands” or Oman or Palau or Panama or Poland or Romania or Russia or “Russian Federation” or Samoa or “Saint Lucia” or “St Lucia” or “Saint Kitts” or “St Kitts” or “Saint Vincent” or “St Vincent” or Serbia or Seychelles or Slovakia or “Slovak Republic” or “South Africa” or Turkey or Uruguay or Venezuela or Yugoslavia) 9. TI (“American Samoa” or Argentina or Belize or Botswana or Brazil or Bulgaria or Chile or Comoros or “Costa Rica” or Croatia or Dominica or Guinea or Gabon or Grenada or Grenadines or Hungary or Kazakhstan or Latvia or Lebanon or Libia or libyan or Libya or Lithuania or Malaysia or Mauritius or Mayotte or Mexico or Micronesia or Montenegro or Nevis or “Northern Mariana Islands” or Oman or Palau or Panama or Poland or Romania or Russia or “Russian Federation” or Samoa or “Saint Lucia” or “St Lucia” or “Saint Kitts” or “St Kitts” or “Saint Vincent” or “St Vincent” or Serbia or Seychelles or Slovakia or “Slovak Republic” or “South Africa” or Turkey or Uruguay or Venezuela or Yugoslavia) 10. AB (“American Samoa” or Argentina or Belize or Botswana or Brazil or Bulgaria or Chile or Comoros or “Costa Rica” or Croatia or Dominica or Guinea or Gabon or Grenada or Grenadines or Hungary or Kazakhstan or Latvia or Lebanon or Libia or libyan or Libya or Lithuania or Malaysia or Mauritius or Mayotte or Mexico or Micronesia or Montenegro or Nevis or “Northern Mariana Islands” or Oman or Palau or Panama or Poland or Romania or Russia or “Russian Federation” or Samoa or “Saint Lucia” or “St Lucia” or “Saint Kitts” or “St Kitts” or “Saint Vincent” or “St Vincent” or Serbia or Seychelles or Slovakia or “Slovak Republic” or “South Africa” or Turkey or Uruguay or Venezuela or Yugoslavia) 11. TI (Africa or Asia or “South America” or “Latin America” or “Central America”) 12. AB (Africa or Asia or “South America” or “Latin America” or “Central America”) 13. (SU “Asia+”) 14. (SU “West Indies+”) 15. (SU “South America+”) 16. (SU “Latin America”) 17. (SU “Central America+”) 18. (SU “Africa+”) 19. (SU “Developing Countries”)  or/1-19  1. (SU "Juvenile Delinquency") 2. AB (juvenile N1 delinquen*) 3. AB (school N1 violence) 4. (SU "Juvenile Offenders+") 5. (SU "[Child Behavior Disorders](http://web.ebscohost.com/ehost/mesh/tree?term=Child%20Behavior%20Disorders&sid=a3e3919d-8eb9-4b24-8097-5c21bd819813%40sessionmgr110&vid=15)")  or/21-2520 and 26  1. SU ("Crime") 2. SU ("Aggression") 3. SU ("Bullying") 4. SU ("Violence") 5. (SU "Violence") 6. (SU "Homicide") 7. AB (conduct N1 problem*) 8. AB (behavio#r N1 problem*) 9. AB (disruptive N1 behavio#r) 10. AB (conduct N1 disorder*) 11. AB (behavio#r N1 disorder*) 12. AB (aggressive N1 behavio#r) 13. AB (aggression) 14. AB (aggressive) 15. AB (antisocial N1 behavio#r) 16. AB (anti-social N1 behavio#r) 17. AB (gang) 18. AB (gangs) 19. AB (criminal N1 behavio#r) 20. AB (violent N1 crime) 21. AB (homicid*) 22. AB (violence) 23. AB (violent) 24. AB (crime) 25. AB (crimes) 26. AB (criminal*) 27. AB (bully) 28. AB (bullying) 29. AB (delinquent*)  \|  \| \| --- \|  1. AB (delinquenc*) 2. TX (oppositional N1 defiant N1 disorder*) 3. TX (disruptive N1 behavio#r N1 disorder*) 4. AB (externalizing N1 behavio#r N1 problem*) 5. AB (externalizing) 6. AB (externalising) 7. AB (externalized) 8. AB (externalised) 9. AB (externaliz*) 10. AB (externalis*) 11. AB (externalizing N1 behavio#r) 12. AB (externalising N1 behavio#r) 13. or /28-69 14. 20 AND 70 15. (SU " Child+") 16. (SU "Adolescence") 17. AB (Adolescen*) 18. AB (Adolescence) 19. AB (Adolescent) 20. AB (adolescents) 21. AB (Child*) 22. AB (child) 23. AB (children) 24. AB (childhood) 25. AB (youth*) 26. AB (youth) 27. AB (youths) 28. AB (student*) 29. AB (Students) 30. AB (Student) 31. AB (teen*) 32. AB (teenager) 33. AB (teenagers) 34. AB (boy*) 35. AB (boy) 36. AB (boys) 37. AB (girl*) 38. AB (girl) 39. AB (girls) 40. AB (pupil) 41. AB (pupils) 42. AB (pupil*) 43. AB (youngster*) 44. AB (youngster) 45. AB (youngsters) 46. AB (juvenile*) 47. AB (juvenile) 48. AB (juveniles) 49. AB (young N1 adult*) 50. AB (infant*) 51. AB (infants) 52. AB (infant) 53. AB (baby*) 54. AB (baby) 55. AB (babies) 56. AB (toddler) 57. AB (toddler*) 58. AB (toddlers) 59. or/72-115 60. 20 and 116 | 124 |
| **Sociological Abstracts**  **+**  **Social Services Abstracts**  **(ProQuest)** | 1. ab(Africa or Asia or "Latin America" or "South America" or Caribbean or "West Indies" or "Eastern Europe" or Soviet or Arab or "Middle East" or "Latin America" or "Central America") OR (ab(Afghanistan or Albania or Algeria or Angola or Antigua or Barbuda or Argentina or Armenia or Armenian or Aruba or Azerbaijan or Bahrain or Bangladesh or Barbados or Benin or Byelarus or Byelorussian or Belarus or Belorussian or Belorussia or Belize or Bhutan or Bolivia or Bosnia or Herzegovina or Hercegovina or Botswana or Brasil or Brazil or Bulgaria or Burkina Faso or Burkina Fasso or Upper Volta or Burundi or Urundi or Cambodia or Khmer Republic or Kampuchea or Cameroon or Cameroons or Cameron or Camerons or Cape Verde or Central African Republic or Chad or Chile or China or Colombia or Comoros or Comoro Islands or Comores or Mayotte or Congo or Zaire or Costa Rica or Cote d'Ivoire or Ivory Coast or Croatia or Cuba or Cyprus or Czechoslovakia or Czech Republic or Slovakia or Slovak Republic or Djibouti or French Somaliland or Dominica or Dominican Republic or East Timor or East Timur or Timor Leste or Ecuador or Egypt or United Arab Republic or El Salvador or Eritrea or Estonia or Ethiopia or Fiji or Gabon or Gabonese Republic or Gambia or Gaza or Georgia Republic or Georgian Republic or Ghana or Gold Coast or Greece or Grenada or Guatemala or Guinea or Guam or Guiana or Guyana or Haiti or Honduras or Hungary or India or Maldives or Indonesia or Iran or Iraq or Isle of Man or Jamaica or Jordan or Kazakhstan or Kazakh or Kenya or Kiribati or Korea or Kosovo or Kyrgyzstan or Kirghizia or Kyrgyz Republic or Kirghiz or Kirgizstan or Lao PDR or Laos or Latvia or Lebanon or Lesotho or Basutoland or Liberia or Libya or Lithuania or Macedonia or Madagascar or Malagasy Republic or Malaysia or Malaya or Malay or Sabah or Sarawak or Malawi or Nyasaland or Mali or Malta or Marshall Islands or Mauritania or Mauritius or Agalega Islands or Mexico or Micronesia or Middle East or Moldova or Moldovia or Moldovian or Mongolia or Montenegro or Morocco or Ifni or Mozambique or Myanmar or Myanma or Burma or Namibia or Nepal or Netherlands Antilles or New Caledonia or Nicaragua or Niger or Nigeria or Northern Mariana Islands or Oman or Muscat or Pakistan or Palau or Palestine or Panama or Paraguay or Peru or Philippines or Philipines or Phillipines or Phillippines or Poland or Portugal or Puerto Rico or Romania or Rumania or Roumania or Russia or Russian or Rwanda or Ruanda or Saint Kitts or St Kitts or Nevis or Saint Lucia or St Lucia or Saint Vincent or St Vincent or Grenadines or Samoa or Samoan Islands or Navigator Island or Navigator Islands or Sao Tome or Saudi Arabia or Senegal or Serbia or Montenegro or Seychelles or Sierra Leone or Slovenia or Sri Lanka or Ceylon or Solomon Islands or Somalia or South Africa or Sudan or Suriname or Surinam or Swaziland or Syria or Tajikistan or Tadzhikistan or Tadjikistan or Tadzhik or Tanzania or Thailand or Togo or Togolese Republic or Tonga or Trinidad or Tobago or Tunisia or Turkey or Turkmenistan or Turkmen or Uganda or Ukraine or Uruguay or USSR or Soviet Union or Union of Soviet Socialist Republics or Uzbekistan or Uzbek or Vanuatu or New Hebrides or Venezuela or Vietnam or Viet Nam or West Bank or Yemen or Yugoslavia or Zambia or Zimbabwe or Rhodesia)) OR (AB “Developing Countries”) OR (ab(developing NEAR/1 world)) OR (ab(poor* NEAR/1 nation*)) OR (ab(developing NEAR/1 countr*)) OR (ab(developing NEAR/1 region*)) OR (ab(third NEAR/1 world)) OR SU.EXACT.EXPLODE("[Developing Countries](http://libsta28.lib.cam.ac.uk:2086/professional/thesaurus/browsepage.thesaurusbrowse.termscontainer.thesaurustermrelationalview.thesaurustermlink:browsethesaurusview/2/2786/updateZone_0?site=eric)") 2. AB(delinquent*) OR AB(delinquenc*) OR AB(school NEAR/1 violence) OR AB(juvenile NEAR/1 delinquency) OR AB(juvenile NEAR/1 delinquent) OR AB(juvenile NEAR/1 delinquents) OR SU.exact("JUVENILE DELINQUENCY") OR SU.exact("DELINQUENCY") OR SU.exact("JUVENILE OFFENDERS") 3. 1 and 2 4. (SU.EXACT.EXPLODE("Crime")) OR (SU.EXACT.EXPLODE("Aggression")) OR (SU.EXACT.EXPLODE("Behavior Problems")) OR (SU.EXACT.EXPLODE("Violence")) OR (SU.EXACT.EXPLODE("Gangs")) OR (ab(gang*)) OR (ab(conduct NEAR/1 problem*)) OR (ab(behavio*r NEAR/1 problem*)) OR (ab(conduct NEAR/1 disorder*)) OR (ab(antisocial NEAR/1 behavio*r*)) OR (ab(oppositional NEAR/1 defiant NEAR/1 disorder*)) OR (AB "Aggression") OR (AB "Social Behavior Disorders") OR (AB "Crime") OR (AB "Violence") OR (AB "Homicide") OR (AB "Assault and Battery") OR (AB "Aggression") OR (AB(conduct NEAR/1 problem*)) OR (AB(behavio#r NEAR/1 problem*)) OR (AB(disruptive NEAR/1 behavio#r)) OR (AB(conduct NEAR/1 disorder*)) OR (AB(behavio#r NEAR/1 disorder*)) OR (AB(aggressive NEAR/1 behavio#r)) OR (AB(aggression) OR AB(aggressive)) OR (AB(antisocial NEAR/1 behavio#r)) OR (AB(anti-social NEAR/1 behavio#r)) OR (AB(gang)) OR (AB(gangs)) OR (AB(criminal N1 behavio#r)) OR (AB(violent NEAR/1 crime)) OR (AB(homicid*)) OR (AB(violence)) OR (AB(violent)) OR (AB(crime)) OR (AB(crimes)) OR (AB(criminal*)) OR (AB(bully)) OR (AB(bullying)) OR TX (oppositional N1 defiant N1 disorder*) OR TX (disruptive N1 behavio#r N1 disorder*) 5. (SU.EXACT.EXPLODE("Adolescents")) OR (SU.EXACT.EXPLODE("Infants")) OR (SU.EXACT.EXPLODE("Children")) OR (AB "Adolescence") OR AB(Adolescen*) OR AB(Adolescence) OR AB(Adolescent) OR AB(adolescents) OR AB(Child*) OR AB(child) OR AB(children) OR AB(childhood) OR AB(youth*) OR AB(youth) OR AB(youths) OR AB(student*) OR AB(Students) OR AB(Student) OR AB(teen*) OR AB(teenager) OR AB(teenagers) OR AB(boy*) OR AB(boy) OR AB(boys) OR AB(girl*) OR AB(girl) OR AB(girls) OR AB(pupil) OR AB(pupils) OR AB(pupil*) OR AB(youngster*) OR AB(youngster) OR AB(youngsters) OR AB(juvenile*) OR AB(juvenile) OR AB(juveniles) OR AB(young NEAR/1 adult*) OR AB(infant*) OR AB(infants) OR AB(infant) OR AB(baby*) OR AB(baby) OR AB(babies) OR AB(toddler) OR AB(toddler*) OR AB(toddlers)’ 6. 4 and 5 7. 1 and 6 | 3404 |
| **Applied Social Sciences Index and fAbstracts (ProQuest)** | 1. (ab(Africa or Asia or "Latin America" or "South America" or Caribbean or "West Indies" or "Eastern Europe" or Soviet or Arab or "Middle East" or "Latin America" or "Central America")) OR (ab(Afghanistan or Albania or Algeria or Angola or Antigua or Barbuda or Argentina or Armenia or Armenian or Aruba or Azerbaijan or Bahrain or Bangladesh or Barbados or Benin or Byelarus or Byelorussian or Belarus or Belorussian or Belorussia or Belize or Bhutan or Bolivia or Bosnia or Herzegovina or Hercegovina or Botswana or Brasil or Brazil or Bulgaria or Burkina Faso or Burkina Fasso or Upper Volta or Burundi or Urundi or Cambodia or Khmer Republic or Kampuchea or Cameroon or Cameroons or Cameron or Camerons or Cape Verde or Central African Republic or Chad or Chile or China or Colombia or Comoros or Comoro Islands or Comores or Mayotte or Congo or Zaire or Costa Rica or Cote d'Ivoire or Ivory Coast or Croatia or Cuba or Cyprus or Czechoslovakia or Czech Republic or Slovakia or Slovak Republic or Djibouti or French Somaliland or Dominica or Dominican Republic or East Timor or East Timur or Timor Leste or Ecuador or Egypt or United Arab Republic or El Salvador or Eritrea or Estonia or Ethiopia or Fiji or Gabon or Gabonese Republic or Gambia or Gaza or Georgia Republic or Georgian Republic or Ghana or Gold Coast or Greece or Grenada or Guatemala or Guinea or Guam or Guiana or Guyana or Haiti or Honduras or Hungary or India or Maldives or Indonesia or Iran or Iraq or Isle of Man or Jamaica or Jordan or Kazakhstan or Kazakh or Kenya or Kiribati or Korea or Kosovo or Kyrgyzstan or Kirghizia or Kyrgyz Republic or Kirghiz or Kirgizstan or Lao PDR or Laos or Latvia or Lebanon or Lesotho or Basutoland or Liberia or Libya or Lithuania or Macedonia or Madagascar or Malagasy Republic or Malaysia or Malaya or Malay or Sabah or Sarawak or Malawi or Nyasaland or Mali or Malta or Marshall Islands or Mauritania or Mauritius or Agalega Islands or Mexico or Micronesia or Middle East or Moldova or Moldovia or Moldovian or Mongolia or Montenegro or Morocco or Ifni or Mozambique or Myanmar or Myanma or Burma or Namibia or Nepal or Netherlands Antilles or New Caledonia or Nicaragua or Niger or Nigeria or Northern Mariana Islands or Oman or Muscat or Pakistan or Palau or Palestine or Panama or Paraguay or Peru or Philippines or Philipines or Phillipines or Phillippines or Poland or Portugal or Puerto Rico or Romania or Rumania or Roumania or Russia or Russian or Rwanda or Ruanda or Saint Kitts or St Kitts or Nevis or Saint Lucia or St Lucia or Saint Vincent or St Vincent or Grenadines or Samoa or Samoan Islands or Navigator Island or Navigator Islands or Sao Tome or Saudi Arabia or Senegal or Serbia or Montenegro or Seychelles or Sierra Leone or Slovenia or Sri Lanka or Ceylon or Solomon Islands or Somalia or South Africa or Sudan or Suriname or Surinam or Swaziland or Syria or Tajikistan or Tadzhikistan or Tadjikistan or Tadzhik or Tanzania or Thailand or Togo or Togolese Republic or Tonga or Trinidad or Tobago or Tunisia or Turkey or Turkmenistan or Turkmen or Uganda or Ukraine or Uruguay or USSR or Soviet Union or Union of Soviet Socialist Republics or Uzbekistan or Uzbek or Vanuatu or New Hebrides or Venezuela or Vietnam or Viet Nam or West Bank or Yemen or Yugoslavia or Zambia or Zimbabwe or Rhodesia)) OR (AB “Developing Countries”) OR (ab(developing NEAR/1 world)) OR (ab(poor* NEAR/1 nation*)) OR (ab(developing NEAR/1 countr*)) OR (ab(developing NEAR/1 region*)) OR (ab(third NEAR/1 world)) OR (SU.EXACT.EXPLODE"[Developing Countries](http://libsta28.lib.cam.ac.uk:2086/professional/thesaurus/browsepage.thesaurusbrowse.termscontainer.thesaurustermrelationalview.thesaurustermlink:browsethesaurusview/2/2786/updateZone_0?site=eric)") 2. (SU.EXACT.EXPLODE("Crime")) OR (SU.EXACT.EXPLODE("Aggression")) OR (SU.EXACT("Bullying")) OR (SU.EXACT.EXPLODE("Violence")) OR (SU.EXACT ("Criminal behaviour")) OR (SU.EXACT ("Oppositional defiant disorder")) OR SU.exact("CONDUCT DISORDERS") OR (ab(gang*)) OR (ab(conduct NEAR/1 problem*)) OR (ab(behavio*r NEAR/1 problem*)) OR (ab(conduct NEAR/1 disorder*)) OR (ab(antisocial NEAR/1 behavio*r*)) OR (ab(oppositional NEAR/1 defiant NEAR/1 disorder*)) OR (AB "Aggression") OR (AB "Social Behavior Disorders") OR (AB "Crime") OR (AB "Violence") OR (AB "Homicide") OR (AB "Assault and Battery") OR (AB "Aggression") OR (AB(conduct NEAR/1 problem*)) OR (AB(behavio#r NEAR/1 problem*)) OR (AB(disruptive NEAR/1 behavio#r)) OR (AB(conduct NEAR/1 disorder*)) OR (AB(behavio#r NEAR/1 disorder*)) OR (AB(aggressive NEAR/1 behavio#r)) OR (AB(aggression) OR AB(aggressive)) OR (AB(antisocial NEAR/1 behavio#r)) OR (AB(anti-social NEAR/1 behavio#r)) OR (AB(gang)) OR (AB(gangs)) OR (AB(criminal N1 behavio#r)) OR (AB(violent NEAR/1 crime)) OR (AB(homicid*)) OR (AB(violence)) OR (AB(violent)) OR (AB(crime)) OR (AB(crimes)) OR (AB(criminal*)) OR (AB(bully)) OR (AB(bullying)) OR TX (oppositional N1 defiant N1 disorder*) OR TX (disruptive N1 behavio#r N1 disorder*) 3. AB(delinquent*) OR AB(delinquenc*) OR AB(school NEAR/1 violence) OR AB(juvenile NEAR/1 delinquency) OR AB(juvenile NEAR/1 delinquent) OR AB(juvenile NEAR/1 delinquents) OR SU.exact("JUVENILE DELINQUENCY") OR SU.exact("DELINQUENCY") OR SU.exact("JUVENILE OFFENDERS") 4. **(**SU.EXACT.EXPLODE"Children") OR (SU.EXACT.EXPLODE("Adolescence")) OR (SU.EXACT.EXPLODE("Youth")) OR (AB "Adolescence") OR AB(Adolescen*) OR AB(Adolescence) OR AB(Adolescent) OR AB(adolescents) OR AB(Child*) OR AB(child) OR AB(children) OR AB(childhood) OR AB(youth*) OR AB(youth) OR AB(youths) OR AB(student*) OR AB(Students) OR AB(Student) OR AB(teen*) OR AB(teenager) OR AB(teenagers) OR AB(boy*) OR AB(boy) OR AB(boys) OR AB(girl*) OR AB(girl) OR AB(girls) OR AB(pupil) OR AB(pupils) OR AB(pupil*) OR AB(youngster*) OR AB(youngster) OR AB(youngsters) OR AB(juvenile*) OR AB(juvenile) OR AB(juveniles) OR AB(young NEAR/1 adult*) OR AB(infant*) OR AB(infants) OR AB(infant) OR AB(baby*) OR AB(baby) OR AB(babies) OR AB(toddler) OR AB(toddler*) OR AB(toddlers) | 801 |
| **International Bibliography of the Social Sciences (IBSS) (ProQuest)** | 1. (ab(Africa or Asia or "Latin America" or "South America" or Caribbean or "West Indies" or "Eastern Europe" or Soviet or Arab or "Middle East" or "Latin America" or "Central America")) OR (ab(Afghanistan or Albania or Algeria or Angola or Antigua or Barbuda or Argentina or Armenia or Armenian or Aruba or Azerbaijan or Bahrain or Bangladesh or Barbados or Benin or Byelarus or Byelorussian or Belarus or Belorussian or Belorussia or Belize or Bhutan or Bolivia or Bosnia or Herzegovina or Hercegovina or Botswana or Brasil or Brazil or Bulgaria or Burkina Faso or Burkina Fasso or Upper Volta or Burundi or Urundi or Cambodia or Khmer Republic or Kampuchea or Cameroon or Cameroons or Cameron or Camerons or Cape Verde or Central African Republic or Chad or Chile or China or Colombia or Comoros or Comoro Islands or Comores or Mayotte or Congo or Zaire or Costa Rica or Cote d'Ivoire or Ivory Coast or Croatia or Cuba or Cyprus or Czechoslovakia or Czech Republic or Slovakia or Slovak Republic or Djibouti or French Somaliland or Dominica or Dominican Republic or East Timor or East Timur or Timor Leste or Ecuador or Egypt or United Arab Republic or El Salvador or Eritrea or Estonia or Ethiopia or Fiji or Gabon or Gabonese Republic or Gambia or Gaza or Georgia Republic or Georgian Republic or Ghana or Gold Coast or Greece or Grenada or Guatemala or Guinea or Guam or Guiana or Guyana or Haiti or Honduras or Hungary or India or Maldives or Indonesia or Iran or Iraq or Isle of Man or Jamaica or Jordan or Kazakhstan or Kazakh or Kenya or Kiribati or Korea or Kosovo or Kyrgyzstan or Kirghizia or Kyrgyz Republic or Kirghiz or Kirgizstan or Lao PDR or Laos or Latvia or Lebanon or Lesotho or Basutoland or Liberia or Libya or Lithuania or Macedonia or Madagascar or Malagasy Republic or Malaysia or Malaya or Malay or Sabah or Sarawak or Malawi or Nyasaland or Mali or Malta or Marshall Islands or Mauritania or Mauritius or Agalega Islands or Mexico or Micronesia or Middle East or Moldova or Moldovia or Moldovian or Mongolia or Montenegro or Morocco or Ifni or Mozambique or Myanmar or Myanma or Burma or Namibia or Nepal or Netherlands Antilles or New Caledonia or Nicaragua or Niger or Nigeria or Northern Mariana Islands or Oman or Muscat or Pakistan or Palau or Palestine or Panama or Paraguay or Peru or Philippines or Philipines or Phillipines or Phillippines or Poland or Portugal or Puerto Rico or Romania or Rumania or Roumania or Russia or Russian or Rwanda or Ruanda or Saint Kitts or St Kitts or Nevis or Saint Lucia or St Lucia or Saint Vincent or St Vincent or Grenadines or Samoa or Samoan Islands or Navigator Island or Navigator Islands or Sao Tome or Saudi Arabia or Senegal or Serbia or Montenegro or Seychelles or Sierra Leone or Slovenia or Sri Lanka or Ceylon or Solomon Islands or Somalia or South Africa or Sudan or Suriname or Surinam or Swaziland or Syria or Tajikistan or Tadzhikistan or Tadjikistan or Tadzhik or Tanzania or Thailand or Togo or Togolese Republic or Tonga or Trinidad or Tobago or Tunisia or Turkey or Turkmenistan or Turkmen or Uganda or Ukraine or Uruguay or USSR or Soviet Union or Union of Soviet Socialist Republics or Uzbekistan or Uzbek or Vanuatu or New Hebrides or Venezuela or Vietnam or Viet Nam or West Bank or Yemen or Yugoslavia or Zambia or Zimbabwe or Rhodesia)) OR (AB “Developing Countries”) OR (ab(developing NEAR/1 world)) OR (ab(poor* NEAR/1 nation*)) OR (ab(developing NEAR/1 countr*)) OR (ab(developing NEAR/1 region*)) OR (ab(third NEAR/1 world)) OR (SU.EXACT.EXPLODE("[Developing Countries](http://libsta28.lib.cam.ac.uk:2086/professional/thesaurus/browsepage.thesaurusbrowse.termscontainer.thesaurustermrelationalview.thesaurustermlink:browsethesaurusview/2/2786/updateZone_0?site=eric)")) 2. (SU.EXACT.EXPLODE("Crime")) OR (SU.EXACT.EXPLODE("Aggression")) OR (SU.EXACT.EXPLODE("Bullying")) OR (SU.EXACT ("Violence")) OR (SU.EXACT.EXPLODE("Gang")) OR (SU.EXACT.EXPLODE("Crime")) OR (SU.EXACT.EXPLODE("Aggression")) OR (SU.EXACT.EXPLODE("Bullying")) OR (SU.EXACT.EXPLODE("Violence")) OR (ab(gang*)) OR (ab(conduct NEAR/1 problem*)) OR (ab(behavio*r NEAR/1 problem*)) OR (ab(conduct NEAR/1 disorder*)) OR (ab(antisocial NEAR/1 behavio*r*)) OR (ab(oppositional NEAR/1 defiant NEAR/1 disorder*)) OR (AB "Aggression") OR (AB "Social Behavior Disorders") OR (AB "Crime") OR (AB "Violence") OR (AB "Homicide") OR (AB "Assault and Battery") OR (AB "Aggression") OR (AB(conduct NEAR/1 problem*)) OR (AB(behavio#r NEAR/1 problem*)) OR (AB(disruptive NEAR/1 behavio#r)) OR (AB(conduct NEAR/1 disorder*)) OR (AB(behavio#r NEAR/1 disorder*)) OR (AB(aggressive NEAR/1 behavio#r)) OR (AB(aggression) OR AB(aggressive)) OR (AB(antisocial NEAR/1 behavio#r)) OR (AB(anti-social NEAR/1 behavio#r)) OR (AB(gang)) OR (AB(gangs)) OR (AB(criminal N1 behavio#r)) OR (AB(violent NEAR/1 crime)) OR (AB(homicid*)) OR (AB(violence)) OR (AB(violent)) OR (AB(crime)) OR (AB(crimes)) OR (AB(criminal*)) OR (AB(bully)) OR (AB(bullying)) 3. AB(delinquent*) OR AB(delinquenc*) OR TX (oppositional N1 defiant N1 disorder*) OR TX (disruptive N1 behavio#r N1 disorder*) OR AB(school NEAR/1 violence) OR AB(juvenile NEAR/1 delinquency) OR AB(juvenile NEAR/1 delinquent) OR AB(juvenile NEAR/1 delinquents) 4. **(**SU.EXACT.EXPLODE("Children")) OR (SU.EXACT.EXPLODE("Adolescence")) OR (SU.EXACT.EXPLODE("Youth")) OR (AB "Adolescence") OR AB(Adolescen*) OR AB(Adolescence) OR AB(Adolescent) OR AB(adolescents) OR AB(Child*) OR AB(child) OR AB(children) OR AB(childhood) OR AB(youth*) OR AB(youth) OR AB(youths) OR AB(student*) OR AB(Students) OR AB(Student) OR AB(teen*) OR AB(teenager) OR AB(teenagers) OR AB(boy*) OR AB(boy) OR AB(boys) OR AB(girl*) OR AB(girl) OR AB(girls) OR AB(pupil) OR AB(pupils) OR AB(pupil*) OR AB(youngster*) OR AB(youngster) OR AB(youngsters) OR AB(juvenile*) OR AB(juvenile) OR AB(juveniles) OR AB(young NEAR/1 adult*) OR AB(infant*) OR AB(infants) OR AB(infant) OR AB(baby*) OR AB(baby) OR AB(babies) OR AB(toddler) OR AB(toddler*) OR AB(toddlers) | 1687 |
| **ERIC (ProQuest)** | 1. (ab(Africa or Asia or "Latin America" or "South America" or Caribbean or "West Indies" or "Eastern Europe" or Soviet or Arab or "Middle East" or "Latin America" or "Central America")) OR (ab(Afghanistan or Albania or Algeria or Angola or Antigua or Barbuda or Argentina or Armenia or Armenian or Aruba or Azerbaijan or Bahrain or Bangladesh or Barbados or Benin or Byelarus or Byelorussian or Belarus or Belorussian or Belorussia or Belize or Bhutan or Bolivia or Bosnia or Herzegovina or Hercegovina or Botswana or Brasil or Brazil or Bulgaria or Burkina Faso or Burkina Fasso or Upper Volta or Burundi or Urundi or Cambodia or Khmer Republic or Kampuchea or Cameroon or Cameroons or Cameron or Camerons or Cape Verde or Central African Republic or Chad or Chile or China or Colombia or Comoros or Comoro Islands or Comores or Mayotte or Congo or Zaire or Costa Rica or Cote d'Ivoire or Ivory Coast or Croatia or Cuba or Cyprus or Czechoslovakia or Czech Republic or Slovakia or Slovak Republic or Djibouti or French Somaliland or Dominica or Dominican Republic or East Timor or East Timur or Timor Leste or Ecuador or Egypt or United Arab Republic or El Salvador or Eritrea or Estonia or Ethiopia or Fiji or Gabon or Gabonese Republic or Gambia or Gaza or Georgia Republic or Georgian Republic or Ghana or Gold Coast or Greece or Grenada or Guatemala or Guinea or Guam or Guiana or Guyana or Haiti or Honduras or Hungary or India or Maldives or Indonesia or Iran or Iraq or Isle of Man or Jamaica or Jordan or Kazakhstan or Kazakh or Kenya or Kiribati or Korea or Kosovo or Kyrgyzstan or Kirghizia or Kyrgyz Republic or Kirghiz or Kirgizstan or Lao PDR or Laos or Latvia or Lebanon or Lesotho or Basutoland or Liberia or Libya or Lithuania or Macedonia or Madagascar or Malagasy Republic or Malaysia or Malaya or Malay or Sabah or Sarawak or Malawi or Nyasaland or Mali or Malta or Marshall Islands or Mauritania or Mauritius or Agalega Islands or Mexico or Micronesia or Middle East or Moldova or Moldovia or Moldovian or Mongolia or Montenegro or Morocco or Ifni or Mozambique or Myanmar or Myanma or Burma or Namibia or Nepal or Netherlands Antilles or New Caledonia or Nicaragua or Niger or Nigeria or Northern Mariana Islands or Oman or Muscat or Pakistan or Palau or Palestine or Panama or Paraguay or Peru or Philippines or Philipines or Phillipines or Phillippines or Poland or Portugal or Puerto Rico or Romania or Rumania or Roumania or Russia or Russian or Rwanda or Ruanda or Saint Kitts or St Kitts or Nevis or Saint Lucia or St Lucia or Saint Vincent or St Vincent or Grenadines or Samoa or Samoan Islands or Navigator Island or Navigator Islands or Sao Tome or Saudi Arabia or Senegal or Serbia or Montenegro or Seychelles or Sierra Leone or Slovenia or Sri Lanka or Ceylon or Solomon Islands or Somalia or South Africa or Sudan or Suriname or Surinam or Swaziland or Syria or Tajikistan or Tadzhikistan or Tadjikistan or Tadzhik or Tanzania or Thailand or Togo or Togolese Republic or Tonga or Trinidad or Tobago or Tunisia or Turkey or Turkmenistan or Turkmen or Uganda or Ukraine or Uruguay or USSR or Soviet Union or Union of Soviet Socialist Republics or Uzbekistan or Uzbek or Vanuatu or New Hebrides or Venezuela or Vietnam or Viet Nam or West Bank or Yemen or Yugoslavia or Zambia or Zimbabwe or Rhodesia)) OR (AB “Developing Countries”) OR (SU.EXACT.EXPLODE("[Developing Countries](http://libsta28.lib.cam.ac.uk:2086/professional/thesaurus/browsepage.thesaurusbrowse.termscontainer.thesaurustermrelationalview.thesaurustermlink:browsethesaurusview/2/2786/updateZone_0?site=eric)")) OR (ab(developing NEAR/1 world)) OR (ab(poor* NEAR/1 nation*)) OR (ab(developing NEAR/1 countr*)) OR (ab(developing NEAR/1 region*)) OR (ab(third NEAR/1 world)**)** OR (SU.EXACT.EXPLODE("Foreign Countries")) OR (SU.EXACT.EXPLODE("[Developing Nations](http://libsta28.lib.cam.ac.uk:2086/professional/thesaurus/browsepage.thesaurusbrowse.termscontainer.thesaurustermrelationalview.thesaurustermlink:browsethesaurusview/2/2786/updateZone_0?site=eric)")) 2. AB(delinquent*) OR AB(delinquenc*) OR TX (oppositional N1 defiant N1 disorder*) OR TX (disruptive N1 behavio#r N1 disorder*) OR AB(school NEAR/1 violence) OR AB(juvenile NEAR/1 delinquency) OR AB(juvenile NEAR/1 delinquent) OR AB(juvenile NEAR/1 delinquents) 3. 1 and 2 4. (SU.EXACT.EXPLODE("Crime")) OR (SU.EXACT.EXPLODE("Aggression")) OR (SU.EXACT.EXPLODE("Bullying")) OR (SU.EXACT.EXPLODE("Violence")) OR (ab(gang*)) OR (ab(conduct NEAR/1 problem*)) OR (ab(behavio*r NEAR/1 problem*)) OR (ab(conduct NEAR/1 disorder*)) OR (ab(antisocial NEAR/1 behavio*r*)) OR (ab(oppositional NEAR/1 defiant NEAR/1 disorder*)) OR (AB "Aggression") OR (AB "Social Behavior Disorders") OR (AB "Crime") OR (AB "Violence") OR (AB "Homicide") OR (AB "Assault and Battery") OR (AB "Aggression") OR (AB(conduct NEAR/1 problem*)) OR (AB(behavio#r NEAR/1 problem*)) OR (AB(disruptive NEAR/1 behavio#r)) OR (AB(conduct NEAR/1 disorder*)) OR (AB(behavio#r NEAR/1 disorder*)) OR (AB(aggressive NEAR/1 behavio#r)) OR (AB(aggression) OR AB(aggressive)) OR (AB(antisocial NEAR/1 behavio#r)) OR (AB(anti-social NEAR/1 behavio#r)) OR (AB(gang)) OR (AB(gangs)) OR (AB(criminal N1 behavio#r)) OR (AB(violent NEAR/1 crime)) OR (AB(homicid*)) OR (AB(violence)) OR (AB(violent)) OR (AB(crime)) OR (AB(crimes)) OR (AB(criminal*)) OR (AB(bully)) OR (AB(bullying)) 5. **(**SU.EXACT.EXPLODE("Adolescents")) OR (SU.EXACT.EXPLODE("Early Adolescents")) OR (SU.EXACT.EXPLODE("Children")) OR (SU.EXACT.EXPLODE("Youth")) OR (SU.EXACT.EXPLODE("Late Adolescents")) OR (AB "Adolescence") OR AB(Adolescen*) OR AB(Adolescence) OR AB(Adolescent) OR AB(adolescents) OR AB(Child*) OR AB(child) OR AB(children) OR AB(childhood) OR AB(youth*) OR AB(youth) OR AB(youths) OR AB(student*) OR AB(Students) OR AB(Student) OR AB(teen*) OR AB(teenager) OR AB(teenagers) OR AB(boy*) OR AB(boy) OR AB(boys) OR AB(girl*) OR AB(girl) OR AB(girls) OR AB(pupil) OR AB(pupils) OR AB(pupil*) OR AB(youngster*) OR AB(youngster) OR AB(youngsters) OR AB(juvenile*) OR AB(juvenile) OR AB(juveniles) OR AB(young NEAR/1 adult*) OR AB(infant*) OR AB(infants) OR AB(infant) OR AB(baby*) OR AB(baby) OR AB(babies) OR AB(toddler) OR AB(toddler*) OR AB(toddlers) 6. 4 and 5 7. 1 and 6 | 1325 |
| [**National Criminal Justice Reference Service Abstracts Database**](https://www.ncjrs.gov/App/search/thesaurussearch.aspx) | “Developing Countries” | 91 |
| **Web of Science** | 1. Topic=(infants) 2. Topic=(infant) 3. Topic=(Infant*) 4. Topic=(juveniles) 5. Topic=(juvenile) 6. Topic=(juvenile*) 7. Topic=(youngsters) 8. Topic=(youngster) 9. Topic=(youngster*) 10. Topic=(pupil*) 11. Topic=(pupils) 12. Topic=(pupil) 13. Topic=(girls) 14. Topic=(girl) 15. Topic=(boys) 16. Topic=(boy) 17. Topic=(teenagers) 18. Topic=(teenager) 19. Topic=(teen*) 20. Topic=(students) 21. Topic=(student) 22. Topic=(student*) 23. Topic=(youths) 24. Topic=(youth) 25. Topic=(youth*) 26. Topic=(childhood) 27. Topic=(childhood*) 28. Topic=(children*) 29. Topic=(child) 30. Topic=(child*) 31. Topic=(adolescence) 32. Topic=(adolescents) 33. Topic=(adolescent) 34. Topic=(adolescen*) 35. Topic=(toddlers) 36. Topic=(toddler) 37. Topic=(babies) 38. Topic=(baby) 39. Topic=(young NEAR/1 adult*) 40. Or/1-39 41. Topic=(externalis*) 42. Topic=(externaliz*) 43. Topic=(externalised) 44. Topic=(externalized) 45. Topic=(externalising) 46. Topic=(externalizing) 47. Topic=(bully) 48. Topic=(bullying) 49. Topic=(bully*) 50. Topic=(criminal NEAR/1 behavio$r*) 51. Topic=(criminal*) 52. Topic=(crimes) 53. Topic=(crime) 54. Topic=(violent NEAR/1 crime*) 55. Topic=(aggressive NEAR/1 behavio$r*) 56. Topic=(anti-social) 57. Topic=(antisocial) 58. Topic=(aggressive) 59. Topic=(aggress*) 60. Topic=(aggression) 61. Topic=(antisocial NEAR/1 behavio$r*) 62. Topic=(disruptive NEAR/1 behavio$r NEAR/1 disorder*) 63. Topic=(oppositional NEAR/1 defiant NEAR/1 disorder*) 64. Topic=(behavio$r NEAR/1 disorder*) 65. Topic=(behavio$r NEAR/1 problem*) 66. Topic=(conduct NEAR/1 disorder*) 67. Topic=(conduct NEAR/1 problem*) 68. Topic=(gangs) 69. Topic=(gang) 70. Topic=(homicide*) 71. Topic=(violen*) 72. Topic=(violence) 73. Topic=(violent) 74. Or/41-73 75. Topic=(school NEAR/1 violence) 76. Topic=(juvenile NEAR/1 delinquent) 77. Topic=(juvenile NEAR/1 delinquency) 78. Or/ 75-77 79. Topic=(deprived NEAR/1 (countr* OR nation*)) 80. Topic=((“less developed”) NEAR/1 (countr* OR nation*)) 81. Topic=((“under developed”) NEAR/1 (countr* OR nation*)) 82. Topic=((“low income”) NEAR/1 (economy or economies)) 83. Topic=((“under developed”) NEAR/1 (economy or economies)) 84. Topic=((“middle income”) NEAR/1 (economy or economies)) 85. Topic=((“under developed”) NEAR/1 (economy or economies)) 86. Topic=(“less developed” NEAR/1 (economy or economies)) 87. Topic=((“under developed”) NEAR/1 (economy or economies)) 88. Topic=(underdeveloped NEAR/1 (economy or economies)) 89. Topic=((poor) NEAR/1 (countr* OR nation*)) 90. Topic=((developing NEAR/1 nation*)) 91. Topic=((developing NEAR/1 region*)) 92. Topic=((developing NEAR/1 countr*)) 93. Topic=((developing NEAR/1 world)) 94. Topic=((developing) NEAR/1 (economy or economies)) 95. Topic=(third NEAR/1 world) 96. Topic=(Afghanistan or Albania or Algeria or Angola or Antigua or Barbuda or Argentina or Armenia or Armenian or Aruba or Azerbaijan or Bahrain or Bangladesh or Barbados or Benin or Byelarus or Byelorussian or Belarus or Belorussian or Belorussia or Belize or Bhutan or Bolivia or Bosnia or Herzegovina or Hercegovina or Botswana or Brasil or Brazil or Bulgaria or Burkina Faso or Burkina Fasso or Upper Volta or Burundi or Urundi or Cambodia or Khmer Republic or Kampuchea or Cameroon or Cameroons or Cameron or Camerons or Cape Verde or Central African Republic or Chad or Chile or China or Colombia or Comoros or Comoro Islands or Comores or Mayotte or Congo or Zaire or Costa Rica or Cote d'Ivoire or Ivory Coast or Croatia or Cuba or Cyprus or Czechoslovakia or Czech Republic or Slovakia or Slovak Republic or Djibouti or French Somaliland or Dominica or Dominican Republic or East Timor or East Timur or Timor Leste or Ecuador or Egypt or United Arab Republic or El Salvador or Eritrea or Estonia or Ethiopia or Fiji or Gabon or Gabonese Republic or Gambia or Gaza or Georgia Republic or Georgian Republic or Ghana or Gold Coast or Greece or Grenada or Guatemala or Guinea or Guam or Guiana or Guyana or Haiti or Honduras or Hungary or India or Maldives or Indonesia or Iran or Iraq or Isle of Man or Jamaica or Jordan or Kazakhstan or Kazakh or Kenya or Kiribati or Korea or Kosovo or Kyrgyzstan or Kirghizia or Kyrgyz Republic or Kirghiz or Kirgizstan or Lao PDR or Laos or Latvia or Lebanon or Lesotho or Basutoland or Liberia or Libya or Lithuania or Macedonia or Madagascar or Malagasy Republic or Malaysia or Malaya or Malay or Sabah or Sarawak or Malawi or Nyasaland or Mali or Malta or Marshall Islands or Mauritania or Mauritius or Agalega Islands or Mexico or Micronesia or Middle East or Moldova or Moldovia or Moldovian or Mongolia or Montenegro or Morocco or Ifni or Mozambique or Myanmar or Myanma or Burma or Namibia or Nepal or Netherlands Antilles or New Caledonia or Nicaragua or Niger or Nigeria or Northern Mariana Islands or Oman or Muscat or Pakistan or Palau or Palestine or Panama or Paraguay or Peru or Philippines or Philipines or Phillipines or Phillippines or Poland or Portugal or Puerto Rico or Romania or Rumania or Roumania or Russia or Russian or Rwanda or Ruanda or Saint Kitts or St Kitts or Nevis or Saint Lucia or St Lucia or Saint Vincent or St Vincent or Grenadines or Samoa or Samoan Islands or Navigator Island or Navigator Islands or Sao Tome or Saudi Arabia or Senegal or Serbia or Montenegro or Seychelles or Sierra Leone or Slovenia or Sri Lanka or Ceylon or Solomon Islands or Somalia or South Africa or Sudan or Suriname or Surinam or Swaziland or Syria or Tajikistan or Tadzhikistan or Tadjikistan or Tadzhik or Tanzania or Thailand or Togo or Togolese Republic or Tonga or Trinidad or Tobago or Tunisia or Turkey or Turkmenistan or Turkmen or Uganda or Ukraine or Uruguay or USSR or Soviet Union or Union of Soviet Socialist Republics or Uzbekistan or Uzbek or Vanuatu or New Hebrides or Venezuela or Vietnam or Viet Nam or West Bank or Yemen or Yugoslavia or Zambia or Zimbabwe or Rhodesia) 97. Topic=(Africa or "Latin America" or "South America" or Caribbean or "West Indies" or "Eastern Europe" or Soviet or Arab or "Middle East" or "Latin America" or "Central America") 98. Or/79-97 99. 40 and 74 100. 99 and 98 101. 78 and 98 | 6248 |
| **JOLIS** (IMF, World Bank and International Finance Corporation) | <http://external.worldbankimflib.org/uhtbin/cgisirsi/?ps=Uvm3MkrFSe/JL/0/49>  (aggression OR violence OR homicide OR gang OR bully OR crime OR “juvenile delinquency” OR “conduct problem” OR “conduct disorder” OR “behavior problem” OR “behavior disorder”)  AND  (adolescent OR child OR youth OR student OR teen OR boy OR girl OR pupil OR youngster OR juvenile OR infant) | 80 |
| **World Bank** | https://openknowledge.worldbank.org/discover?scope=%2F&query=%28aggression+OR+violence+OR+homicide+OR+gang+OR+bully+OR+crime+OR+%E2%80%9Cjuvenile+delinquency%E2%80%9D+OR+%E2%80%9Cconduct+problem%E2%80%9D+OR+%E2%80%9Cconduct+disorder%E2%80%9D+OR+%E2%80%9Cbehavior+problem%E2%80%9D+OR+%E2%80%9Cbehavior+disorder%E2%80%9D%29+AND+%28adolescent+OR+child+OR+youth+OR+student+OR+teen+OR+boy+OR+girl+OR+pupil+OR+youngster+OR+juvenile+OR+infant%29&submit=Go  (aggression OR violence OR homicide OR gang OR bully OR crime OR “juvenile delinquency” OR “conduct problem” OR “conduct disorder” OR “behavior problem” OR “behavior disorder”) AND (adolescent OR child OR youth OR student OR teen OR boy OR girl OR pupil OR youngster OR juvenile OR infant) | 1236 |

**LILACS**

RUN 1

(child OR niño OR criança OR infant OR lactante OR lactente OR Adolescent OR Adolescente OR “Child Psychiatry” OR “Psiquiatría Infantil” OR “Psiquiatria Infantil” OR “Child Behavior” OR “Conducta Infantil” OR “Comportamento Infantil” OR “Adolescent Behavior” OR “Conducta del Adolescente” OR “Comportamento do Adolescente” OR Adolescent Development” OR “Desarrollo del Adolescente” OR “Desenvolvimento do Adolescente” OR “Adolescent Behavior” OR “Conducta del Adolescente” OR “Comportamento do Adolescente”)

[Subject descriptor]

AND

gang OR gangs OR pandilla OR quadrilha OR crimes OR criminal OR Crimen OR Crime OR (antisocial AND behavio$r) OR antisocial OR anti-social OR “antisocial behavio$r” OR “anti-social behavior” OR “comportamento anti-social” OR “conducta anti-social” OR violen$ OR Violencia OR Violência OR violence OR violent OR violen$ OR bully$ OR “Acoso Escolar” OR Bullying OR aggress$ OR aggression OR Agresión OR Agressão OR Homicidio OR Homicídio OR Acoso Escolar OR bullying OR domestic violence OR Violencia Doméstica OR Violência Doméstica OR conducta antisocial

[Words]

370

RUN 2

child OR children OR adolescent OR Adolescente OR child$ OR adolescen$ OR youth$ OR student$ OR teen$ OR boy$ OR girl$ OR pupil$ OR youngster$ OR juvenile$ OR infant$ OR infan$ OR baby OR babies OR preschool OR preschool$ OR criança OR infant OR infants OR lactante OR lactente OR neonat$ OR baby OR babies OR kid OR kids OR toddler$ OR jóvenes OR niña OR niño OR criança OR newborn

[Words]

AND

“Domestic Violence” OR “Violencia Doméstica” OR “Violência Doméstica” OR “Social Behavior Disorders” OR “Trastorno de la Conducta Social” OR “Transtornos do Comportamento Social” OR aggression OR Agresión OR Agressão OR Homicide OR Homicidio OR Homicídio OR bully OR “Acoso Escolar” OR Bullying OR “oppositional defiant disorder” OR “trastorno desafiante por oposición” OR “transtorno desafiador de oposição” OR “conduct disorder” OR “Trastorno del Comportamiento” OR “Transtorno da Conduta” OR “transtorno desafiador-opositivo” OR “conducta antisocial” or “transtorno da conduta” OR “transtorno da personalidade anti-social” OR “Transtornos do

Comportamento”

[Subject descriptor]

228

RUN 3

child OR niño OR criança OR infant OR lactante OR lactente OR Adolescent OR Adolescente OR “Child Psychiatry” OR “Psiquiatría Infantil” OR “Psiquiatria Infantil” OR “Child Behavior” OR “Conducta Infantil” OR “Comportamento Infantil” OR “Adolescent Behavior” OR “Conducta del Adolescente” OR “Comportamento do Adolescente” OR Adolescent Development” OR “Desarrollo del Adolescente” OR “Desenvolvimento do Adolescente” OR “Adolescent Behavior” OR “Conducta del Adolescente” OR “Comportamento do Adolescente” OR “Adolescent Psychiatry” OR “Psiquiatría del Adolescente” OR “Psiquiatria do Adolescente”

[Subject descriptor]

AND

“Domestic Violence” OR “Violencia Doméstica” OR “Violência Doméstica” OR “Social Behavior Disorders” OR “Trastorno de la Conducta Social” OR “Transtornos do Comportamento Social” OR aggression OR Agresión OR Agressão OR Homicide OR Homicidio OR Homicídio OR bully OR “Acoso Escolar” OR Bullying OR “oppositional defiant disorder” OR “trastorno desafiante por oposición” OR “transtorno desafiador de oposição” OR “conduct disorder” OR “Trastorno del Comportamiento” OR “Transtorno da Conduta” OR “transtorno desafiador-opositivo” OR “conducta antisocial” or “transtorno da conduta” OR “transtorno da personalidade anti-social” OR “Transtornos do

Comportamento”

[Subject descriptor]

4538

RUN 4

child OR children OR adolescent OR Adolescente OR child$ OR adolescen$ OR youth$ OR student$ OR teen$ OR boy$ OR girl$ OR pupil$ OR youngster$ OR juvenile$ OR infant$ OR infan$ OR baby OR babies OR preschool OR preschool$ OR criança OR infant OR infants OR lactante OR lactente OR neonat$ OR baby OR babies OR kid OR kids OR toddler$ OR jóvenes OR niña OR niño OR criança OR newborn

[Words]

AND

gang OR gangs OR pandilla OR quadrilha OR crimes OR criminal OR Crimen OR Crime OR antisocial OR anti-social OR “antisocial behavio$r” OR “anti-social behavior” OR “comportamento anti-social” OR “conducta anti-social” OR “conducta antisocial” OR violen$ OR Violencia OR Violência OR violence OR violent OR bully$ OR “Acoso Escolar” OR Bullying OR aggress$ OR aggression OR Agresión OR Agressão OR Homicidio OR Homicídio OR Acoso Escolar OR “domestic violence” OR “Violencia Doméstica” OR “Violência Doméstica”

[Words]

NOT

liposarcoma

473

RUN  5

“Child Behavior Disorders” OR “delinquencia” OR “delinquencia femenina” OR “delinquencia juvenil” or delincuencial or delincuenciales or delincuente or delincuentes OR “juvenile delinquency” OR delincuen$ OR “Delincuencia Juvenil” OR “Delinquência Juvenil” OR “Transtornos do Comportamento Infantil” OR Delinquencia or Delinquen$ or “Trastornos de la Conducta Infantil” or Transtornos do “Comportamento Infantil”

[Words]

**SCIELO**

RUN 1 - 60

child OR niño OR criança OR infant OR lactante OR lactente OR Adolescent OR Adolescente OR child OR children OR adolescent OR child$ OR adolescen$ OR youth$ OR student$ OR teen$ OR boy$ OR girl$ OR pupil$ OR youngster$ OR juvenile$ OR infant$ OR infan$ OR baby OR babies OR preschool OR preschool$ OR criança OR infant OR infants OR lactante OR lactente OR neonat$ OR baby OR babies OR kid OR kids OR toddler$ OR jóvenes OR niña OR niño OR criança OR newborn

[All indexes]

AND

“Acoso Escolar” OR “Violência Doméstica” OR Transtornos do Comportamento OR “Transtornos do Comportamento Social” OR Agressão OR Homicídio OR Bullying OR “transtorno desafiador-opositivo” OR “Transtorno da Conduta” OR “conducta antisocial” or “transtorno da conduta” OR “transtorno da personalidade anti-social”

[Subject descriptor]

RUN 2 - 1189

child OR niño OR criança OR infant OR lactante OR lactente OR Adolescent OR Adolescente OR child OR children OR adolescent OR child$ OR adolescen$ OR youth$ OR student$ OR teen$ OR boy$ OR girl$ OR pupil$ OR youngster$ OR juvenile$ OR infant$ OR infan$ OR baby OR babies OR preschool OR preschool$ OR criança OR infant OR infants OR lactante OR lactente OR neonat$ OR baby OR babies OR kid OR kids OR toddler$ OR jóvenes OR niña OR niño OR criança OR newborn

[All indexes]

AND

gang OR gangs OR pandilla OR quadrilha OR crimes OR criminal OR crimen OR crime OR “comportamento anti-social” OR “conducta anti-social” OR violence OR violen$ OR Violencia OR Violência OR violent OR bully$ OR aggress$ OR aggression OR Agresión OR Agressão OR Homicidio OR Homicídio OR Acoso Escolar OR bullying OR domestic violence OR Violencia Doméstica OR Violência Doméstica OR conducta antisocial OR “Transtorno da Conduta” OR “transtorno desafiador de oposição” OR “transtorno da personalidade anti-social”OR “Transtornos do Comportamento”

[All indexes]

RUN 3- 9

“delinquencia” OR “delinquencia femenina” OR “delinquencia juvenil” or delincuencial or delincuenciales or delincuente or delincuentes OR “Transtornos do Comportamento Infantil”

[Subject descriptor]

RUN 4-106

Delinquencia or Delinquen$ or Transtornos do Comportamento Infantil

[All indexes]
