# Supplementary material for: Methods for conducting systematic reviews of risk factors in low- and middle-income countries
Source: BMC Med Res Methodol. 2016 Mar 15;16:32. doi: 10.1186/s12874-016-0134-2 (PMC4791911; doi:10.1186/s12874-016-0134-2)
Supplement: Additional file 2: — Review protocol. The file includes the protocol for this systematic review, also published at http://www.vrc.crim.cam.ac.uk/vrcresearch/meLMIC/protcolriskfactors. (PDF 748 kb) [file 12874_2016_134_MOESM2_ESM.pdf]

Risk Factors for Child and Adolescent Conduct Problems and Youth Crime and Violence in Low- and Middle-Income Countries: Two-Part Systematic Review

Review Protocol, 18 September 2013 [Corrected 11 November 2013]

Joseph Murray, Wellcome Trust Research Career Development Fellow and Senior Research Associate, Department of Psychiatry, Cambridge University - Principal Investigator

Yulia Shenderovich - Research Assistant, Department of Psychiatry, Cambridge University

Manuel Eisner, Professor of Comparative & Developmental Criminology, Deputy Director of the Institute of Criminology, Cambridge University - Co Investigator

Maria Ttofi, University Lecturer in Psychological Criminology, Research Fellow, Wolfson College, Cambridge University - Co Investigator

Chris Mikton, Technical Officer, Violence and Injury Prevention, World Health Organization – Co Investigator

Frances Gardner, Professor of Child and Family Psychology in the Department of Social Policy and Intervention, and Fellow of Wolfson College, Oxford University - Co Investigator

Richard Parker, Institute of Public Health, Cambridge University - Statistical Adviser

Contents

Contents 2

1. Background 3
2. Objectives 6
3. Methods
    - 3.1 Search strategy 6
    - 3.2 Criteria for inclusion and exclusion of studies 6
    - 3.3 Description of methods used in primary research 9
    - 3.4 Effect size measure 9
    - 3.5 Data extraction process 9
    - 3.6 Determination of independent findings 10
    - 3.7 Synthesis of bivariate results 12
    - 3.8 Synthesis of covariate-adjusted results 12
    - 3.9 Statistical procedures and conventions 12
4. Timetable 13
5. Acknowledgements 13
6. Statement of conflicts of interest 13
7. References 14

1. Background

Violence is a major global health issue, recently listed as a priority for the post-2015 development agenda with a call to "reduce violent deaths ... and eliminate all forms of violence against children" (UN, 2013: 31). Levels of violence (e.g., homicide) in many low- and middle-income countries (LMICs) are much higher than in high-income countries (HICs) (see Table 1). However, most research on causes of violence and other types of antisocial behaviour has been conducted in HICs. This two-part systematic review aims to identify risk factors for both childhood conduct problems and youth crime and violence in LMICs. A risk factor is a characteristic of a person or his/her environment that predicts an increased probability of engaging in antisocial behaviour (Kraemer, Lowe, & Kupfer, 2005).

Table 1. Homicide rates in 2008 and Disability Adjusted Life Years (DALYs) lost from violence in 2004

		Homicides per one hundred thousand	DALY (thousands)
High Income Countries		2.7	886,297
Low- and Middle-Income Countries, by region	Africa	20.1	6,333,294
	The Americas	24.1	6,024,751
	Eastern Mediterranean	3.9	1,346,008
	Europe	9.8	1,826,177
	South-East Asia	5.8	3,444,677
	Western Pacific	2.8	1,775,947

Adapted from World Health Organization Global Burden of Disease tables (http://www.who.int/healthinfo/global_burden_disease/, accessed 9/3/2012). One DALY can be thought of as one year of healthy life lost due to violence.

The World Health Organization (WHO) defines violence as: "The intentional use of physical force or power, threatened or actual, against oneself, another person, or against a group or community, that either results in or has a high likelihood of resulting in injury, death, psychological harm, maldevelopment or deprivation" (WHO, 2002: 5). In the landmark *World Report on Violence and Health* (WHO, 2002) three broad types of violence are distinguished: self-directed violence, interpersonal violence, collective violence. The current systematic review will focus on interpersonal violence, which includes both violence between family members and violence between individuals in the community.

Violence is one type of antisocial behaviour. Most violent offenders also engage in other types of antisocial acts (Farrington & Loeber 2000) and most antisocial adults have a history of childhood conduct disorders (Loeber et al. 1993; Moffitt 1993). Conduct disorders are characterized by a "repetitive and persistent pattern of dissocial, aggressive, or defiant conduct" in a child (WHO, 2004: 209). The Diagnostic and Statistical Manual of Mental Disorders (DSM), specifies oppositional defiant disorder (ODD), which is represented by social or educational impairment and a pattern of negative, hostile, and defiant behaviour that does not violate the law, and conduct disorder (CD), which is characterized by behaviours violating the rights of others and age-appropriate social norms (American Psychiatric Association, 1994).

Childhood conduct problems, such as CD and ODD, are among the most common child mental health problems, experienced by 5–8% of children (Moffitt & Scott, 2008). Conduct problems are related to lifelong adverse outcomes, such as criminal violence, school failure, unemployment, physical health problems and substance abuse (Fergusson, Horwood, & Ridder, 2005; Odgers et al., 2007; Colman et al., 2009). When assessed in a longitudinal study of children from a disadvantaged borough in inner London, the costs of public services for individuals with conduct disorder were estimated to be ten times higher than for those with no conduct problems (Scott et al., 2001).

Several extensive reviews are available of risk factors for conduct problems and violence in HICs (for examples, see Hawkins et al., 1998; Farrington & Welsh, 2007; Murray & Farrington, 2010). Reviews typically concentrate on longitudinal studies, investigating individual, family, peer and social risk factors (see Figure 1). Although 90% of the world's 2.2 billion children live in LMICs (UNICEF, 2008), and significantly more children live in the poorest parts of the world than ever before (You, Anthony, Wardlaw, & Jenkins, 2013), little evidence about their mental health is available (Kieling et al. 2011). To our knowledge, no systematic review has been conducted of risk factors for antisocial behaviour specifically in LMICs. While previous reviews did not explicitly exclude research from LMICs, they did not actively search in other languages, had relatively exclusive methodological eligibility criteria and might not have captured some of the more recent research in LMICs. It cannot be assumed that risk factors in HICs will replicate elsewhere, or that all important causes of antisocial behaviour in LMICs have been identified in prior reviews. For instance, effects of malnutrition on antisocial behaviour has not been examined in HICs, but in a cohort study based in Mauritius, malnutrition at age three years was a risk factor for aggression up to age 18, controlling for psychosocial adversity (Liu, Raine, Venables, & Mednick, 2004). Although transporting behavioural measures and psychiatric concepts across cultural contexts has limitations (Bird 1996; López & Guarnaccia, 2000; Rescorla et al., 2007; Canino et al., 2010, Goodman et al., 2012), it is important to synthesise available knowledge about risk factors in LMICs to identify potential targets for intervention.

Systematic reviews have become an increasingly common method for assessing effectiveness of social and medical interventions (Davies, 2000; Lavis et al., 2005), offering an important tool in systematically synthesizing and critically appraising evidence (Petrosino 2003). Systematic reviews can also be used to summarize evidence about risk factors (Murray, Farrington, & Eisner, 2009). To design effective interventions, it is necessary to develop etiological theories and identify risk factors for targeted problems (Hawkins et al., 1998; Blum & Ireland, 2004; Piquero et al., 2009); identifying modifiable causal risk factors is especially important. Causal risk factors "are the 'gold' of risk estimation—they can be used both to identify those of high risk of the outcome and to provide the bases for interventions to prevent the outcome" (Kraemer et al. 2005: 32–33). Researchers can move towards identifying causal risk factors by controlling for other variables that might explain risk factor associations (using matching techniques or statistical modelling), using genetically sensitive research designs, exploiting natural experiments, and by investigating within-individual change in antisocial behaviour from before to after exposure to a risk factor and (Murray, Farrington, & Eisner, 2009; Jaffee, Strait, & Odgers, 2012). Protective factors are beyond the scope of the review, but it is important to acknowledge that there may be interactions between risk and protective factors (Jessor 2003).

Figure 1. Overview of some of key risk factors for conduct problems and crime, identified in previous reviews

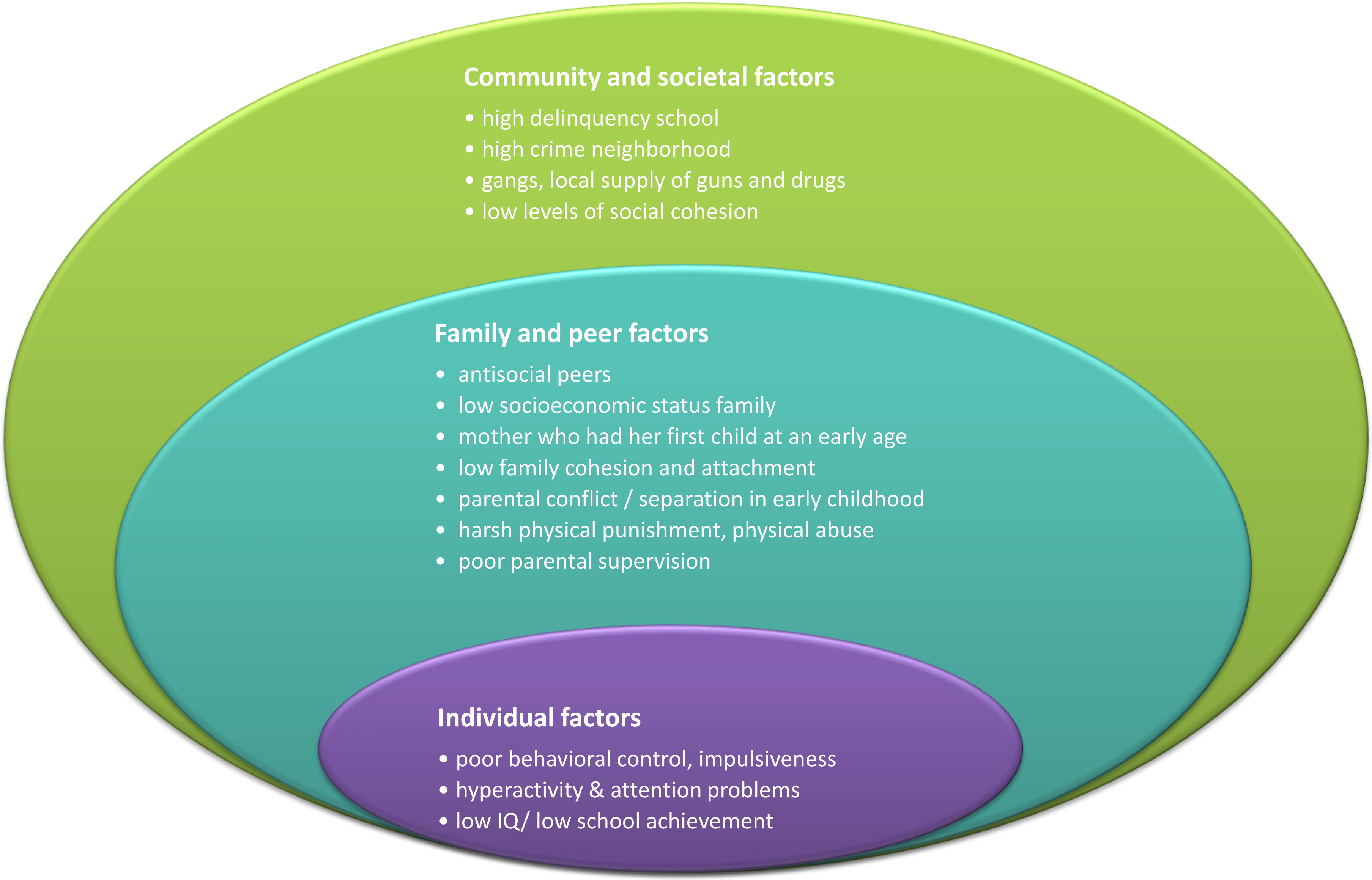

2. Objectives

The primary objectives of the review are to synthesise evidence on the following questions:

1) What are the risk factors for occurrence of child and adolescent conduct problems in LMICs?
2) What are the risk factors for perpetration of youth crime and violence in LMICs?
3) How do risk factors in LMICs compare to well-established risk factors identified in HICs?

As a secondary objective, if sufficient data are available, the review will investigate how the associations between risk factors and outcomes differ according to pre-specified moderators, such as study features and country characteristics (see Section 3.7).

Table 2. Scope of the review process

**Problem**	child and adolescent conduct problems, aggression, conduct disorder, oppositional defiant disorder; youth violence and crime
**Population**	low- and middle-income countries
**Outcome**	standardized measures of conduct problems; self reports, other reports and official records of youth violence and crime
**Study design**	cross-sectional surveys, case-control studies, cohort studies,

3.1 Search strategy

This systematic review will be conducted in line with the Cochrane Handbook guidelines. Anticipating an overlap between studies assessing risk factors for conduct problems and studies assessing risk factors for crime and violence, a single search will be conducted and retrieved studies will be channelled into two separate reviews on these topics (see Figure 2). Studies will be identified through electronic searches of bibliographic databases and grey literature sites, examining citations of retrieved studies, and contacting researchers working in the area. The main searches will be carried out in English, with additional searches in Chinese, Spanish, Portuguese, Russian, Arabic, and French. The importance of searching in non-English language sources is a well-established best practice in systematic reviews (Moher et al., 1996; Egger et al., 1997; Jüni et al., 2002), and especially important for this review focusing on LMICs.

3.2 Criteria for inclusion and exclusion of studies

To be eligible for inclusion in the reviews, the study must meet all the inclusion criteria and none of the exclusion criteria set out in Tables 3 and 4 below.

Table. 4. Inclusion and exclusion criteria for the conduct problems review

Inclusion	Exclusion
Study population:<br>- under 18 years old (children)	
Sampling:<br>- located in a LMIC, defined according to the World Bank during the study publication year<br>- had at least 100 study participants included in the analyses<br>- recruited participants in the community (in households, schools, or maternity hospitals for birth cohort studies) through random, stratified probability, or total sampling; or recruited participants in an institutionalized setting with a matched comparison group in the community	Sampling:<br>- a sample of a specific sub-population (not defined by sex or age), such as natural disaster survivors<br>- participants recruited entirely from a single organization (e.g., only one school or only one hospital)<br>- participants recruited entirely in institutionalized settings, such as youth detention centres<br>- sample defined on the basis of participants having committed prior criminal offences, without a control group in a case-control design<br>- citizens of LMIC countries living abroad
Measures:<br>- measured conduct problems, bullying, ODD or CD, gang membership, or aggression (including subtypes of aggression and bullying) based on a validated measure, such as the Child Behavior Checklist, the Strengths and Difficulties Questionnaire, the Development and Wellbeing Assessment.<br>- assessed the association at the level of an individual between at least one specific risk factor and at least one outcome	Measures:<br>- studies that included ADHD or other disorders within a composite measure alongside conduct problems/CD/ODD/aggression<br>- risk factors that are conglomerations of multiple constructs, such as Raine, A., Brennan, P., Mednick, B., & Mednick, S. A. (1996)'s measure of biosocial risk including marital conflict, maternal rejection, family instability, parental crime, neurological problems, and slow motor development.
Study design:<br>- longitudinal study<br>- cross-sectional survey<br>- case-control: comparison of a group with the outcome (e.g. conduct problems) and those without the outcome	Study design:<br>- qualitative report<br>- prevalence study that does not assess risk factors

Table. 4. Inclusion and exclusion criteria for the violence and crime review

Inclusion	Exclusion
Study population:<br>- 10-29 years old (youth)	
Sampling:<br>- located in a LMIC, defined according to the World Bank during the study publication year<br>- had at least 100 study participants included in the analyses<br>- recruited participants in the community (in households, schools, or maternity hospitals for birth cohort studies) through random, stratified probability, or total sampling; or recruited participants in an institutionalized setting with a matched comparison group in the community	Sampling:<br>- a sample of a specific sub-population (not defined by sex or age), such as natural disaster survivors<br>- participants recruited entirely from a single organization (e.g., only one school or only one hospital)<br>- participants recruited entirely in institutionalized settings, such as youth detention centres or mental health clinics<br>- sample defined on the basis of all participants having violence or crime record, without a control group in a case-control design<br>- citizens of LMIC countries living abroad
Measures:<br>- used either a measure of perpetration of violence (including domestic/ intimate partner violence) or non-violent crime, or combined violent and non-violent crime, based on self-reports, criminal records or other reports.<br>- assessed the association at the level of an individual between at least one risk factor and at least one outcome	Measures:<br>- only assessed risk factors for other types of violence, such as suicide (self-directed violence).<br>- risk factors that are conglomerations of multiple constructs, such as Raine, A., Brennan, P., Mednick, B., & Mednick, S. A. (1996)'s measure of biosocial risk including marital conflict, maternal rejection, family instability, parental crime, neurological problems, and slow motor development.
Study design:<br>- longitudinal study<br>- cross-sectional survey estimating the correlation between a risk factor and a conduct problem outcome<br>- case-control: comparison of a group with the outcome (e.g. delinquency) and those without the outcome	Study design:<br>- qualitative report<br>- prevalence study that does not assess risk factors

3.3 Description of methods used in primary research

A preliminary review of literature identified several relevant cohort studies located in Mauritius (Raine, Reynolds, Venables, Mednick, & Farrington, 1998), China (Taylor, Friday, Ren, Weitekamp, & Kerner, 2004), Brazil (Anselmi et al., 2008; Caicedo et al., 2010), South Africa (Barbarin, Richter, & DeWet, 2001), and Jamaica (McCaw-Binns, 2011). For example, the 1993 Pelotas (Brazil) Birth Cohort Study in the south of Brazil included 5,249 live born children in maternity hospitals during 1993 (Anselmi et al., 2012). This study examined associations (risk ratios) between perinatal and age 11 risk factors and conduct problems at age 15 measured using the Strengths and Difficulties Questionnaire. To give another example, Barbarin, Richter, & DeWet (2001) report a longitudinal birth cohort study of 625 black South African children born during a seven-week period in 1990 to women in Soweto-Johannesburg. The study presents zero-order correlations between different forms of victimization and child outcomes, including opposition (measured by a subscale of the Behavior Problem Index) and aggression (measured by subscales of Child Behavior Checklist).

Although longitudinal studies are more informative regarding causality than cross-sectional (single time point) studies, we believe that there are few relevant longitudinal studies in LMICs, so this systematic review will include both cross-sectional, case-control and longitudinal studies. Our preliminary review of literature suggests that the majority of studies in LMICs are cross-sectional. For instance, Ozbay and colleagues (2006) undertook a survey in Turkey's capital Ankara to test Hirschi's Social Bonding Theory, sampling 1,710 high school students. The researchers assessed the adolescents' self-reported delinquency (assault, school delinquency, public disturbance), and the association of the delinquency score with several indicators of social bonding, controlling for background variables. Other studies used a case-control design, such as Ruchkin, Eisemann, and Hägglöf's (1998) survey in the Arkhangelsk region of Russia, comparing the reported parenting practices experienced by 133 subjects from a juvenile correction centre and 108 matched school-children.

3.4 Effect size measure

The odds ratio (OR) is the effect size we will use to represent the strength of association between risk factors and conduct problem or crime/violence outcomes. The OR is often reported for dichotomous outcomes, such as "convicted/not convicted", and can be easily calculated from other statistical information (Lipsey & Wilson, 2001). If studies do not report odds ratios, we will calculate them wherever possible. The OR represents the odds of an outcome in one group exposed to a risk factor divided by the odds of the outcome in a non-exposed group. That is, the OR represents how more or less likely people with a risk factor are to have conduct problems, or commit violence or crime. An OR below 1.0 shows a reduced probability of risk, OR of 1.0 indicates no difference in risk (no association between the risk factor and outcome), and an OR above 1.0 represents an increased risk; an OR equal or greater than 2.0 indicates a strongly increased risk.

3.5 Data extraction process

The data extraction forms for both reviews are available in separate documents. The studies will be coded by two persons.

3.6 Determination of independent findings

Effect sizes within one meta-analysis have to be independent. If studies report multiple findings for a single sample, all eligible findings will be coded, and one result per meta-analysis will be used from each sample as follows:

- Each type of risk factor will be meta-analysed separately. If a single study includes results both for IQ and socioeconomic status, for example, the two results would be included in separate meta-analyses of these two risk factors.
- Each type of outcome will be meta-analysed separately (see Figure 2 for an illustration). If a single study includes results for a risk factor in relation to both conduct disorder and violence, for example, the two results would be included in separate analyses of these outcomes.
- Bivariate and covariate-adjusted effect sizes will be analysed separately
- If studies report multiple results controlling for third variables (covariate-adjusted effect sizes) for the same risk factor and outcome, we will only include the result from the model with the most covariates controlled.
- In longitudinal studies with results from multiple time points, results for short (≤2 years) medium (>2 year to ≤5 years), and long (>5 years) follow-up periods will be coded separately.
- If several results are reported for a single outcome at a single point in time, these will be averaged.
- Any remaining multiple results for a single risk factor and single outcome in a single sample will be averaged to produce one effect size for meta-analysis.

Figure 2. Distribution of outcome measures from included studies

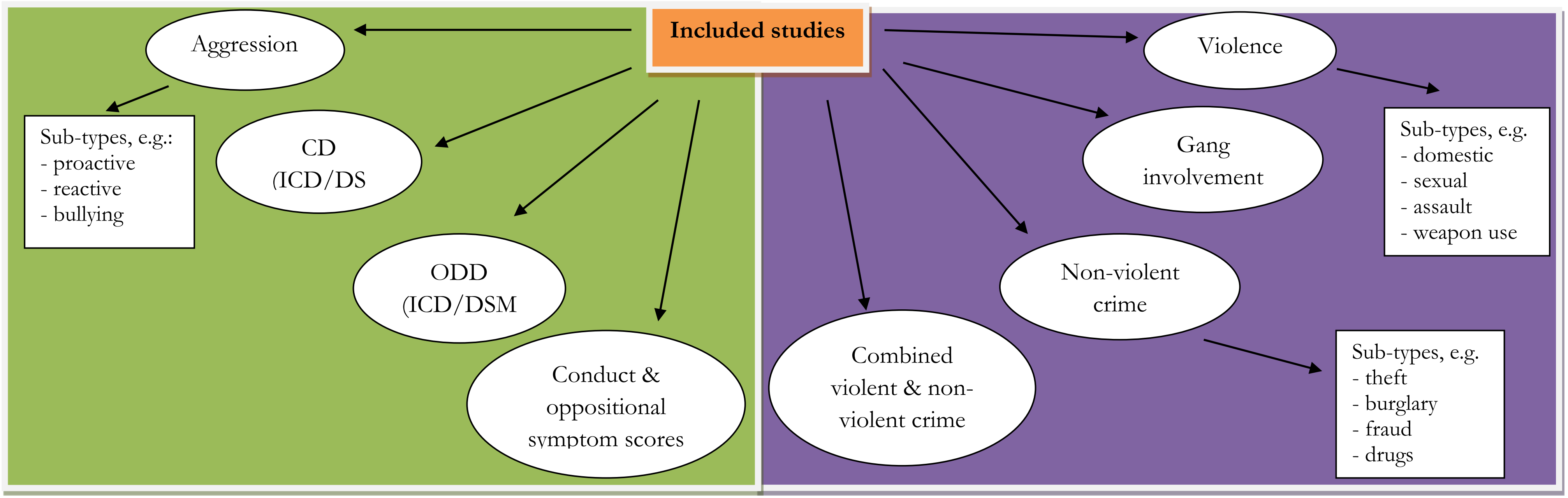

3.7 Synthesis of results

Meta-analyses will be conducted separately of bivariate and covariate-adjusted results. Bi-variate results eligible for the meta-analyses include those from studies in which the participants were matched in terms of age or sex, or when recruitment sites were matched on measures of socioeconomic status. Covariate-adjusted results (e.g. from regression models) will be synthesised separately. Separate meta-analyses will be done for each outcome (and sub-types of outcome) shown in Figure 2, and separately for each risk factor, e.g. for IQ as a risk factor for violence.

In addition to the analyses of main effects, we hope to carry out exploratory moderator analyses to check for differences in risk factor associations according to:
- male/female child
- participant age at outcome
- length of time between risk factor assessment and outcome assessment
- rural/urban setting
- region of the world
- Human Development Index level of the country of study
- national homicide rate
- study design (cross-sectional, case-control, prospective longitudinal)
- outcome measure type
- per cent attrition in longitudinal studies
- covariates controlled for covariate-adjusted effect sizes

However, moderator analyse have weak statistical power and are only possible if sufficient number of studies are available – some suggest at least 10 studies per moderator (Borenstein, Hedges, Higgins, & Rothstein, 2009: 188).

If meta-analysis is not possible, the findings will be narratively summarized.

3.9 Statistical procedures and conventions

Under the fixed effect model in meta-analyses, the assumption is that a single true effect size exists, while the random effects model assumes a distribution of effect sizes (Borenstein, Hedges, Higgins, & Rothstein, 2010). Given the diversity of studies we expect to retrieve, a random-effects model is likely to improve generalizability (Field, 2001). However, in random effects models, small studies with extreme results can have more weight than in fixed effects models (Poole & Greenland, 1999). Therefore, as the Cochrane Handbook recommends, random and fixed-effects estimates will both be computed and compared to check for any major differences.

4. Timetable

Task	*Complete by*
Project planning	July 2013
Testing search strategy	August 2013
Finalizing review protocol	September 2013
Searches in English	September 2013
Searches in other languages	October 2013
Contacting authors for unpublished studies	October 2013
Title inclusion/exclusion	October 2013
Full text inclusion/exclusion	November 2013
Data extraction	December 2013
Data synthesis	February 2013

5. Acknowledgements

The Wellcome Trust is funding this research [089963/Z/09/Z].

6. Statement of conflicts of interest

The authors declare that they have no competing interests.

7. References

American Psychiatric Association (1994). Diagnostic and statistical manual of mental disorders (DSM-IV).

Anselmi, L., Barros, F. C., Teodoro, M. L., Piccinini, C. A., Menezes, A. M. B., Araujo, C. L., & Rohde, L. A. (2008). Continuity of behavioral and emotional problems from pre-school years to pre-adolescence in a developing country. Journal of Child Psychology and Psychiatry, 49(5), 99-507.

Anselmi, L., Menezes, A.M.B., Hallal, P.C., Wehrmeister, F., Goncalves H., Barros, F.C., Murray, J., Rohde, L.A. (2012). Socioeconomic changes and adolescent psychopathology in a Brazilian birth cohort study. J Adolesc Health, 51(6), S5–S10. doi:10.1016/j.jadohealth.2012.06.026

Ashenafi, Y., Kebede, D., Desta, M., Alem, A. (2001). Prevalence of mental and behavioural disorders in Ethiopian children. East Afr Med J, 78(6), 308–311.

Barbarin, O. A., Richter, L. & DeWet, T. (2001). Exposure to violence, coping resources, and psychological adjustment of South African children. *American Journal of Orthopsychiatry*, 71(1), 16-25.

Benjet, Corina, et al. (2009). Youth mental health in a populous city of the developing world: results from the Mexican Adolescent Mental Health Survey. *Journal of Child Psychology and Psychiatry,* 50 (4), 386-395.

Bird, H.R. (1996). Epidemiology of childhood disorders in a crosscultural context. *J Child Psychol Psychiatry*, 37(1), 35–49.

Blum, R. W., & Ireland, M. (2004). Reducing risk, increasing protective factors: findings from the Caribbean Youth Health Survey. *Journal of Adolescent Health*, 35(6), 493-500.

Borenstein, M., Hedges, L. V., Higgins, J. P., & Rothstein, H. R. (2009). *Introduction to meta-analysis.* Wiley.

Borenstein, M., Hedges, L. V., Higgins, J. P. T., & Rothstein, H. R. (2010). A basic introduction to fixed effect and random effects models for meta-analysis. *Research Synthesis Methods*, 1, 97-111. doi: 10.1002/jrsm.12

Brion, M.-J., Victora, C., Matijasevich, A., Horta, B., Anselmi, L., Steer, C., Menezes, A.M.B., Lawlor, D.A., Smith, G. D. (2010). Maternal smoking and child psychological problems: disentangling causal and noncausal effects. *Pediatrics,* 126 (1). doi:10.1542/peds.2009-2754

Caicedo, B., Gonçalves, H., González, D. A., & Victora, C. G. (2010). Violent delinquency in a Brazilian birth cohort: the roles of breast feeding, early poverty and demographic factors. *Paediatric and perinatal epidemiology*, 24(1), 12-23.

Canino, G., Polanczyk, G., Bauermeister, J. J., Rohde, L. A., & Frick, P. J. (2010). Does the prevalence of CD and ODD vary across cultures? *Social psychiatry and psychiatric epidemiology*, 45(7), 695-704.

Colman I, Murray J, Abbott RA, Maughan B, Kuh D, Croudace TJ, Jones PB (2009) Outcomes of conduct problems in adolescence: 40 year follow-up of national cohort. *Br Med, J* 338, 208–211. doi: 10.1136/bmj.a2981

Davies, P. (2000). The relevance of systematic reviews to educational policy and practice. *Oxford Review of Education*, 26(3-4), 365-378.

Deeks, J.J., Higgins, J.P.T., & Altman, D.G. (editors). Chapter 9: Analysing data and undertaking meta-analyses. In: Higgins, J.P.T. & Green, S. (editors). *Cochrane Handbook for Systematic Reviews of Interventions*. Chichester (UK): John Wiley & Sons, 2008.

Egger, M., Zellweger-Zähner, T., Schneider, M., Junker, C., Lengeler, C., & Antes, G. (1997). Language bias in randomised controlled trials published in English and German. *The Lancet*, 350 (9074), 326-329.

Farrington, D.P., & Welsh, B.C. (2007). *Saving children from a life of crime: early risk factors and effective interventions.* Oxford: Oxford University Press.

Fergusson DM, Horwood LJ, Ridder EM. (2005). Show me the child at seven: the consequences of conduct problems in childhood for psychosocial functioning in adulthood. *J Child Psychol Psychiatry*, 46, 837–49.

Gelhorn, H. L., Sakai, J. T., Price, R. K., & Crowley, T. J. (2007). DSM-IV conduct disorder criteria as predictors of antisocial personality disorder. *Comprehensive psychiatry*, 48(6), 529-538.

Goodman, R., Renfrew, D., & Mullick, M. (2000). Predicting type of psychiatric disorder from Strengths and Difficulties Questionnaire (SDQ) scores in child mental health clinics in London and Dhaka. *European child & adolescent psychiatry*, 9(2), 129-134.

Grimshaw, J. (2004). So what has the Cochrane Collaboration ever done for us? A report card on the first 10 years. *Canadian Medical Association Journal*, 171(7), 747-749.

Hawkins, J. D., Herrenkohl, T., Farrington, D. P., Brewer, D., Catalano, R. F., & Harachi, T. W. (1998). A review of predictors of youth violence. In D. P. Farrington & R. Loeber (Eds.), *Serious and violent juvenile offenders: Risk factors and successful interventions*, 106–146. Thousand Oaks, CA: Sage.

Hofman, K., Primack, A., Keusch, G., & Hrynkow, S. (2005). Addressing the growing burden of trauma and injury in low-and middle-income countries. *Journal Information*, *95*(1).

Farrington, D. P., & Loeber, R. (2000). Epidemiology of juvenile violence. *Child and Adolescent Psychiatric Clinics of North America, 9*(4), 733-748.

Field, A.P. (2001). Meta-analysis of correlation coefficients: A Monte Carlo comparison of fixed- and random effects methods. *Psychological Methods*, 6, 161–180.

Jaffee, S. R., Strait, L. B., & Odgers, C. L. (2012). From correlates to causes: Can quasi-experimental studies and statistical innovations bring us closer to identifying the causes of antisocial behavior? *Psychological Bulletin, 138*(2), 272-295. doi: 10.1037/a0026020

Jüni, P., Holenstein, F., Sterne, J., Bartlett, C., & Egger, M. (2002). Direction and impact of language bias in meta-analyses of controlled trials: empirical study. International journal of epidemiology, 31(1), 115-123.

Kieling, C., Baker-Henningham, H., Belfer, M., Conti, G., Ertem, I., Omigbodun, O., Rohde, A, Srinath, S., Ulkuer, N. & Rahman, A. (2011). Child and adolescent mental health worldwide: evidence for action. *The Lancet*, 378 (9801), 1515-1525.

Kraemer, H. C., Lowe, K. K., & Kupfer, D. J. (2005). To your health: How to understand what research tells us about risk. New York, NY: Oxford University Press.

Lavis, J., Davies, H., Oxman, A., Denis, J. L., Golden-Biddle, K., & Ferlie, E. (2005). Towards systematic reviews that inform health care management and policy-making. *Journal of Health Services Research & Policy*, 10 (1), 35-48.

Laub, J. H., & Lauritsen, J. L. (1993). Violent criminal behavior over the life course: A review of the longitudinal and comparative research. *Violence and Victims*, 8(3), 235–252.

Lipsey, M. W., & Wilson, D. B. (2001). *Practical meta-analysis*. Thousand Oaks, CA: Sage.

Liu, J. H., Raine, A., Venables, P. H., & Mednick, S. A. (2004).Malnutrition at age 3 years and externalizing behavior problems at ages 8, 11, and 17 years. *The American Journal of 1093 Psychiatry*, 161(11). http://dx.doi.org/10.1176/appi.ajp.161.11.2005.

Loeber, R., Wung, P., Keenan, K., Giroux, B., Southamer-Loeber, M., van Kammen, W.B. (1993). Developmental pathways in disruptive child behavior. Dev Psychopathol 5(1–2), 101–132. doi:10.1017/S0954579400004296

Loeber, R., Farrington, D.P., Stouthamer-Loeber, M., & Van Kammen, W.B. (1998). *Antisocial behavior and mental health problems: Explanatory factors in childhood and adolescence.* Mahwah, NJ: Lawrence Erlbaum

López, S. R., & Guarnaccia, P. J. (2000). Cultural psychopathology: Uncovering the social world of mental illness. Annual review of psychology, 51(1), 571-598.

McCaw-Binns, A., Ashley, D., Samms-Vaughan, M., Wilks, R., Ferguson, T., Younger, N., Reece, J.-A., Tulloch-Reid, M., & Foster-Williams, K. (2011). Cohort Profile: The Jamaican 1986 Birth Cohort Study. *International journal of epidemiology*, 40(6), 1469-1476.

Moffitt, T.E. (1993). Adolescence-limited and life-course-persistentantisocial behavior: a developmental taxonomy. *Psychol Rev*, 100(4), 674–701. doi:10.1037/0033-295X.100.4.6744.

Moffitt, T.E., Arseneault L, Jaffee SR, Kim-Cohen J, Koenen KC, Odgers CL, Slutske WS,

Moffitt, T. & Scott, S. (2008). Conduct disorders in childhood and adolescence. In *Rutter's Child and Adolescent Psychiatry* (5th edn) (ed M Rutter, D Bishop,D Pine, S Scott, J Stevenson, E Taylor, et al), Blackwell Publishing, 543–64.

Moher, D., P. Fortin, A. R. Jadad, P. Jüni, T. Klassen, J. Le Lorier, A. Liberati, A. Penna, and K. Linde. (1996). Completeness of reporting of trials published in languages other than English: implications for conduct and reporting of systematic reviews. *The Lancet,* 347 (8998), 363-366.

Murray, J., & Farrington, D. P. (2008). The effects of parental imprisonment on children. *Crime and Justice*, 37(1), 133-206.

Murray, J., Farrington, D. P., & Eisner, M. P. (2009). Drawing conclusions about causes from systematic reviews of risk factors: The Cambridge Quality Checklists. *Journal of Experimental Criminology*, 5(1), 1-23.

Murray, J., & Farrington, D. P. (2010). Risk factors for conduct disorder and delinquency: key findings from longitudinal studies. *Canadian Journal of Psychiatry*, 55(10), 633-642.

Murray, J., Anselmi, L., Gallo, E. A. G., Fleitlich-Bilyk, B., & Bordin, I. A. (2013). Epidemiology of childhood conduct problems in Brazil: systematic review and meta-analysis. *Social psychiatry and psychiatric epidemiology*, 1-12.

Odgers, C.L., Caspi, A., Broadbent, J.M., Dickson, N., Hancox, R.J., Harrington, H., Poulton, R., Sears, M.R., Thomson, W.M., & Moffitt, T.E. (2007). Prediction of differential adult health burden by conduct problem subtypes in males. *Arch Gen Psychiatry* 64(4), 476–484. doi:10.1001/archpsyc.64.4.476

Özbay, Ö., & Özcan, Y. Z. (2006). A Test of Hirschi's Social Bonding Theory Juvenile Delinquency in the High Schools of Ankara, Turkey. International Journal of Offender Therapy and Comparative Criminology, 50(6), 711-726.

Panyayong, B., & Wacharasindhu, A. (2002). Psychiatric disorders in Thai school-aged children: II Associated factors. *J Medical Ass Thailand* 85(1), S137–S147

Petrosino, A., Boruch, R. F., Farrington, D. P., Sherman, L. W., & Weisburd, D. (2003). Toward evidence-based criminology and criminal justice: Systematic reviews, the Campbell Collaboration, and the Crime and Justice Group. *International Journal of Comparative Criminology*, 3(1), 42-61.

Piquero, A.R., Farrington, D.P., Welsh, B.C., Tremblay, R.,& Jennings, W.G. (2009). Effects of early family/parent training programs on antisocial behavior and delinquency. *Journal of Experimental Criminology*, *5,* 83–120.

Raine, A., Reynolds, C., Venables, P. H.,Mednick, S. A., & Farrington, D. P. (1998). Fearlessness, stimulation-seeking, and large body size at age 3 years as early predispositions to childhood aggression at age 11 years. Archives of General Psychiatry, 55(8), 745–751. 1151 http://dx.doi.org/10.1001/archpsyc.55.8.745.

Reeves BC, Deeks JJ, Higgins JPT,Wells GA. Chapter 13: Including non-randomized studies. In: Higgins JPT, Green S (editors). (2008). Cochrane Handbook for Systematic Reviews of Interventions. Chichester (UK): John Wiley & Sons.

Rescorla, L., Achenbach, T., Ivanova, M. Y., Dumenci, L., Almqvist, F., Bilenberg, N., Bird, H., & Verhulst, F. (2007). Behavioral and emotional problems reported by parents of children ages 6 to 16 in 31 societies. *Journal of Emotional and behavioral Disorders*, 15(3), 130-142.

Robins, L. N. (1966*). Deviant children grown up: a sociological and psychiatric study of sociopathic personality*. Williams and Wilkins, Baltimore

Ruchkin, V. V., Eisemann, M., & Hägglöf, B. (1998). Parental rearing and problem behaviours in male delinquent adolescents versus controls in northern Russia. *Social psychiatry and psychiatric epidemiology*, 33 (10), 477-482.

Scott S, Knapp M, Henderson J, & Maughan B. (2001). Financial cost of social exclusion: follow up study of antisocial children into adulthood. *Br Med J*, 323, 191–193. doi:10.1136/bmj.323.7306.191

Taylor, T. J., Friday, P. C., Ren, X., Weitekamp, E. G. M., & Kerner, H. J. (2004). Risk and protective factors related to offending: Results from a Chinese cohort study. Australian and New Zealand Journal of Criminology, 37, 13–31.

Ukeje, C. U., & Iwilade, A. (2012). A farewell to innocence? African youth and violence in the twenty-first century. *International Journal of Conflict and Violence,* 6(2), 338-350.

UN (2013). A new global partnership: eradicate poverty and transform economies through sustainable development. The Report of the High-Level Panel of Eminent Persons on the Post-2015 Development Agenda. Retrieved on 20 July 2013 from http://www.un.org/sg/management/pdf/HLP_P2015_Report.pdf

UNICEF. (2008). *Statistics and monitoring.* Retrieved on 31 October 2012 from http://www.unicef.org/statistics.

Viding, E. (2008). Research review: DSM-V conduct disorder: research needs for an evidence base. *J Child Psychol Psychiatry,* 49(1), 3–33. doi:10.1111/j.1469-7610.

Wacharasindhu A, Panyyayong B (2002) Psychiatric disorders in Thai school-aged children: I Prevalence. *J Med Association Thailand*, 85 (1), S125–S136

WHO (2002). World report on violence and health. Retrieved on 10 July 2013 from http://www.who.int/violence_injury_prevention/violence/world_report/en/

WHO. (2004). *ICD-10: International statistical classification of diseases and related health problems.* World Health Organization.

You, D., Anthony, D., Wardlaw, T., & Jenkins, R. (2013). The changing face of global child demographics. The Lancet, 381(9868), 701-703.
