# Supplementary material for: Methods for conducting systematic reviews of risk factors in low- and middle-income countries
Source: BMC Med Res Methodol. 2016 Mar 15;16:32. doi: 10.1186/s12874-016-0134-2 (PMC4791911; doi:10.1186/s12874-016-0134-2)
Supplement: Additional file 3: — Study screening tool. The file includes a detailed screening tool used for selecting studies into the systematic review. (DOCX 31 kb) [file 12874_2016_134_MOESM3_ESM.docx]

S**creening criteria**

|  | **Yes** | **No** | **Only move to next point if the study fulfils the previous criterion. If not, exclude the study.**  **If you are unsure, look for other studies describing the same investigation, or ask someone else for a second opinion.** | | |
| --- | --- | --- | --- | --- | --- |
| **Outcome** |  |  | 1. **Was at least one of these outcomes present within the relevant age group?** Note that studies, in which age and outcomes do not match (e.g., aggression among 10-20 year olds) are not eligible. | | |
|  |  |  | - Aggression - Conduct/externalizing problems* - CD/ODD - Perpetrating bullying   AND   - All participants **0-18 years** old   CHILD REVIEW | - Gang membership - Non-violent crime - Violent & non-violent crime* - Violence (carrying weapons, fighting)   AND   - All participants **10-29 years** old   YOUNG PERSON REVIEW | |
|  | Outcome definitions | | **Aggression** - a measure of child’s physical and/or verbal aggression (can also be termed rage, anger, hostility)  **Conduct/externalizing problems –** CBCL, SDQ, another scale – preferably described in detail – at least enough to ensure the scale assesses externalizing behaviours. Make sure this is not a Total Problem Behaviour Score, which includes externalizing/emotional problems  “delinquent activities such as smoking cigarettes, drinking, lying=cheating, fighting, stealing, breaking things, engaging in public graffiti, and talking back to parents"  Read carefully to make sure that the measure actually reflects what we are looking for. For example, behaviour problems may include internalizing conditions, such as depression, which is not eligible for this review. [not including ADHD or internalizing problems]  (for CD/ODD – standardized measures of conduct or oppositional defiant disorder; for crime and violence there aren’t really widely accepted measures, so we are accepting various measures as long as they seem to describe our constructs of interest).  **CD/ODD –** a DSM/ICD-based diagnosis of conduct or oppositional defiant disorder  **Bullying –** measure of perpetrating bullying. Make sure this does not include victimization from bullying but instead perpetration of bullying | | **Gang membership** – membership in a gang or violent group  **Non-violent crime** – robbery, shoplifting, computer hacking, vehicle theft (but not drug/substance use/abuse)  **Violent & non-violent crime** – for instance, a scale might combine items such as “purposely damaged property, carried a hidden weapon, hit someone with the idea of hurting them”  If the type of crime/delinquency was not specified, and the study was a case-study comparing delinquents and mainstream youth, outcome is classified as violent+non-violent crime  **Violence** – any measure of violence, including fighting and weapon carrying, assault, intimate partner violence |
| **Sampling** |  |  | 2. **Is the study located in a low- or middle-income country**? (defined according to the World Bank as LMIC over 50% of the years 1987-2012)  See list here: <https://docs.google.com/document/d/1wTzKBzg3QDQr5ZO9IIQ_BFTpwDlqXMj9mlKaQiQ65v8/edit?usp=sharing>   1. [check it’s not a sample from LMIC population living in a HIC, e.g. Chinese Americans] | | |
|  |  |  | 3. **Are there at least 100 study participants?** | | |
|  |  |  | 1. 4. **Were the participants recruited through random, stratified probability, or total sampling** in households, schools, communities or maternity hospitals? 2. Ideally, the selection of study sites is also systematic (e.g random selection of schools in the country), but this review only requires systematic selection of individuals **within** study sites (schools/communities). Total sampling may mean recruitment, for instance, of all students in the included schools or all classes of certain age, without any systematic bias. Random sampling may mean random selection of children in schools or neighbourhoods.   For case studies, participants have to be recruited in an institutionalized/specialized setting with a matched comparison group in the community recruited through random, stratified probability, or total sampling within at least one of the groups.  We include studies if sampling is unclear – but make a note of unclear sampling accordingly. However, if it is clear the sampling was by convenience, exclude the paper. | | |
|  |  |  | 5. **Were the participants recruited in more than one organization?**  (e.g. *at least two* schools, hospitals) | | |
| **Study design** |  |  | 6. **Does the paper present a longitudinal, cross-sectional, or case-control study?** (not only a literature review/meta-analysis)  A longitudinal study follows people over time, whereas cross-sectional design measures both risk factor and outcome at the same time. Case-control studies may compare a group of young people with crime/violence record and a group of children without crime/violence record, or a group of children with conduct problems and a group of children without conduct problems. | | |
| **Risk factor** |  |  | **7. Is numeric information (e.g. in prevalence rates, correlation or regression) reported to be able to calculate an effect size for the risk factor (correlate)?**  This may include prevalence rates, e.g. rates of violence in young people who smoke and those who don’t. | | |
|  |  |  | 8**. Is there variation in the presence or level of the risk factor?** This means a non-specialized sample - e.g., if all children in the sample are delinquents, we cannot compare risk factors to non-delinquents. | | |
|  |  |  |  | | |

If you prefer an alternative organization, these same criteria are listed below in a different format:

Inclusion and exclusion criteria for the conduct problems review

| Inclusion | Exclusion |
| --- | --- |
| Study population:  - under 18 years old (children)  Sampling:  - located in a LMIC, defined according to the World Bank during the study publication year  - had at least 100 study participants included in the analyses  - recruited participants in the community (in households, schools, or maternity hospitals for birth cohort studies) through random, stratified probability, or total sampling; or recruited participants in an institutionalized setting with a matched comparison group in the community  Measures:  - measured conduct problems, bullying, ODD or CD, gang membership, or aggression (including subtypes of aggression and bullying) based on a validated measure, such as the Child Behavior Checklist, the Strengths and Difficulties Questionnaire, the Development and Wellbeing Assessment.  - assessed the association at the level of an individual between at least one specific risk factor and at least one outcome  Study design:  - longitudinal study  - cross-sectional survey  - case-control: comparison of a group with the outcome (e.g. conduct problems) and those without the outcome | Sampling: - a sample of a specific sub-population (not defined by sex or age), such as natural disaster survivors - participants recruited entirely from a single organization (e.g., only one school or only one hospital) - participants recruited entirely in institutionalized settings, such as youth detention centres - sample defined on the basis of participants having committed prior criminal offences, without a control group in a case-control design - citizens of LMIC countries living abroad Measures: - studies that included ADHD or other disorders within a composite measure alongside conduct problems/CD/ODD/aggression - risk factors that are conglomerations of multiple constructs, such as Raine, A., Brennan, P., Mednick, B., & Mednick, S. A. (1996)’s measure of biosocial risk including marital conflict, maternal rejection, family instability, parental crime, neurological problems, and slow motor development. Study design: - qualitative report - prevalence study that does not assess risk factors |

Inclusion and exclusion criteria for the violence and crime review

| Inclusion | Exclusion |
| --- | --- |
| Study population: - 10-29 years old (youth) Sampling: - located in a LMIC, defined according to the World Bank during the study publication year - had at least 100 study participants included in the analyses - recruited participants in the community (in households, schools, or maternity hospitals for birth cohort studies) through random, stratified probability, or total sampling; or recruited participants in an institutionalized setting with a matched comparison group in the community Measures: - used either a measure of perpetration of violence (including domestic/ intimate partner violence) or non-violent crime, or combined violent and non-violent crime, based on self-reports, criminal records or other reports. - assessed the association at the level of an individual between at least one risk factor and at least one outcome Study design: - longitudinal study - cross-sectional survey estimating the correlation between a risk factor and a conduct problem outcome - case-control: comparison of a group with the outcome (e.g. delinquency) and those without the outcome | Sampling: - a sample of a specific sub-population (not defined by sex or age), such as natural disaster survivors - participants recruited entirely from a single organization (e.g., only one school or only one hospital) - participants recruited entirely in institutionalized settings, such as youth detention centres or mental health clinics - sample defined on the basis of all participants having violence or crime record, without a control group in a case-control design - citizens of LMIC countries living abroad Measures: - only assessed risk factors for other types of violence, such as suicide (self-directed violence). - risk factors that are conglomerations of multiple constructs, such as Raine, A., Brennan, P., Mednick, B., & Mednick, S. A. (1996)’s measure of biosocial risk including marital conflict, maternal rejection, family instability, parental crime, neurological problems, and slow motor development. Study design: - qualitative report - prevalence study that does not assess risk factors |
